# Activin-Like Kinase 2 Functions in Peri-implantation Uterine Signaling in Mice and Humans

**DOI:** 10.1371/journal.pgen.1003863

**Published:** 2013-11-14

**Authors:** Caterina Clementi, Swamy K. Tripurani, Michael J. Large, Mark A. Edson, Chad J. Creighton, Shannon M. Hawkins, Ertug Kovanci, Vesa Kaartinen, John P. Lydon, Stephanie A. Pangas, Francesco J. DeMayo, Martin M. Matzuk

**Affiliations:** 1 Program in Developmental Biology, Baylor College of Medicine, Houston, Texas, United States of America; 2 Departments of Pathology & Immunology, Baylor College of Medicine, Houston, Texas, United States of America; 3 Center for Reproductive Medicine, Baylor College of Medicine, Houston, Texas, United States of America; 4 Molecular and Cellular Biology, Baylor College of Medicine, Houston, Texas, United States of America; 5 Dan L. Duncan Cancer Center, Baylor College of Medicine, Houston, Texas, United States of America; 6 Obstetrics & Gynecology, Baylor College of Medicine, Houston, Texas, United States of America; 7 Department of Biologic & Materials Sciences, University of Michigan, Ann Arbor, Michigan, United States of America; 8 Center for Drug Discovery, Baylor College of Medicine, Houston, Texas, United States of America; 9 Departments of Molecular and Human Genetics, Baylor College of Medicine, Houston, Texas, United States of America; 10 Departments of Pharmacology, Baylor College of Medicine, Houston, Texas, United States of America; Stanford University School of Medicine, United States of America

## Abstract

Implantation of a blastocyst in the uterus is a multistep process tightly controlled by an intricate regulatory network of interconnected ovarian, uterine, and embryonic factors. Bone morphogenetic protein (BMP) ligands and receptors are expressed in the uterus of pregnant mice, and BMP2 has been shown to be a key regulator of implantation. In this study, we investigated the roles of the BMP type 1 receptor, activin-like kinase 2 (ALK2), during mouse pregnancy by producing mice carrying a conditional ablation of *Alk2* in the uterus (*Alk2* cKO mice). In the absence of ALK2, embryos demonstrate delayed invasion into the uterine epithelium and stroma, and upon implantation, stromal cells fail to undergo uterine decidualization, resulting in sterility. Mechanistically, microarray analysis revealed that CCAAT/enhancer-binding protein β (*Cebpb*) expression is suppressed during decidualization in *Alk2* cKO females. These findings and the similar phenotypes of *Cebpb* cKO and *Alk2* cKO mice lead to the hypothesis that BMPs act upstream of CEBPB in the stroma to regulate decidualization. To test this hypothesis, we knocked down *ALK2* in human uterine stromal cells (hESC) and discovered that ablation of *ALK2* alters hESC decidualization and suppresses *CEBPB* mRNA and protein levels. Chromatin immunoprecipitation (ChIP) analysis of decidualizing hESC confirmed that BMP signaling proteins, SMAD1/5, directly regulate expression of *CEBPB* by binding a distinct regulatory sequence in the 3′ UTR of this gene; CEBPB, in turn, regulates the expression of progesterone receptor (*PGR*). Our work clarifies the conserved mechanisms through which BMPs regulate peri-implantation in rodents and primates and, for the first time, uncovers a linear pathway of BMP signaling through ALK2 to regulate *CEBPB* and, subsequently, *PGR* during decidualization.

## Author Summary

A couple is defined as infertile when failing to become pregnant after one year of regular, unprotected intercourse. Infertility affects more than 10% of couples. The implantation of the embryo in the uterus is one of the most critical steps of pregnancy, and it has been estimated that 75% of pregnancy fails because of peri-implantation defects. An intricate network of molecular pathways regulates the peri-implantation process. It is known that the bone morphogenetic protein (BMP) pathways are part of this network, and herein we investigated how one of the BMP signaling receptors interacts with other factors in the uterus. Our results show an essential and conserved role of this BMP receptor during the implantation of the embryo in mice and humans. Furthermore, we discovered that BMPs act in a linear pathway upstream of two other key regulators of implantation, CEBPB and PGR.

## Introduction

The tunica mucosa of the uterus, called the endometrium, must undergo significant changes to become receptive to implantation of the blastocyst. The endometrium consists of glandular and luminal epithelium and stroma, and when the endometrium is receptive, the embryos can attach to the endometrial epithelium and invade into the stromal compartment. Stromal cells respond to the invasion of the embryo with a wave of proliferation followed by differentiation; this morphological and functional transformation is called decidualization [1]. These steps are fundamental to the implantation process and are dependent upon the action of ovarian progesterone (P4) signaling through its cognate receptor (reviewed in [2]). Several functions have been attributed to the decidua: 1) it provides the growing embryo with growth factors and cytokines; 2) it regulates the local immune response at the feto-maternal interface; 3) it maintains tissue homeostasis during trophoblast invasion; 4) it protects the blastocyst from inflammation and reactive oxygen species; and 5) it supports the angiogenic processes necessary to create new vessels for the perfusion and nourishment of the embryo (reviewed in [3]). The development of the embryo and the cycling of the uterus must be synchronized for implantation to occur. This synchrony requires complex cell-specific crosstalk, and although many factors have been shown to be involved in implantation, it is still unclear how these factors function and interact. One group of signaling proteins that is expressed in the uterus during early pregnancy is the bone morphogenic protein (BMP) subfamily of the transforming growth factor β (TGFβ) superfamily. Unraveling the signaling processes regulated by BMPs during embryo implantation is important for the understanding of endometrial health.

BMP2 was identified as one of the factors that failed to exhibit induction in the decidual uterus treated with the PR antagonist, RU486 [4]. Because BMP2 is essential for embryonic development of the mouse, the function of BMP2 in the postnatal uterus was initially unknown [5]. Conditional genetic deletion of *Bmp2* in the uterus was facilitated by the generation of a mouse model that expresses cre recombinase under control of the progesterone receptor (*Pgr*) promoter [6]. This technology has been largely used to study the functions of multiple genes in the mouse uterus ([7]). Mice lacking BMP2 in the uterus (*Pgr^cre/+^ Bmp2^flox/flox^*) are sterile due to the inability to undergo decidualization [8]. Furthermore, deletion of *Bmp2* in the uterus causes the deregulation of downstream targets like the WNT signaling pathway, PGR signaling, and induction of *Ptgs2*. Roles of BMP2 in the decidual reaction have been confirmed in humans [9].

Because BMP2 is one of the master regulators of implantation [8], and other BMPs are expressed in the uterus during the peri-implantation period [10], [11], it is important to determine the receptors that transduce BMP signaling in the regulation of endometrial function. BMP ligands bind heterodimers of type 1 and type 2 serine/threonine kinase receptors. Upon ligand binding, the type 2 receptor phosphorylates and activates the type 1 receptor, which leads to phosphorylation of receptor-regulated SMAD proteins (R-SMADs; i.e., SMAD1, 5 and 8). Activated SMADs form a complex with the common SMAD (C-SMAD), SMAD4, and accumulate in the nucleus to regulate the expression of genes involved in cell growth, cell differentiation, apoptosis, cellular homeostasis, and other cellular functions. BMP ligands bind different receptors in different contexts: the physiological association with a specific receptor depends on both the binding affinity and the actual availability of the ligand and the receptor in a specific environment. While the type 2 receptors are essential for recognizing the ligands, the type 1 receptors determine the specificity of the intracellular response. Three type 1 receptors, activin-like kinase 2 (ALK2; ACVR1), activin-like kinase 3 (ALK3; BMPR1A), and activin-like kinase 6 (ALK6; BMPR1B), are known to mediate BMP signaling, and all three type 1 receptors are expressed in the pregnant uterus (data not shown). Our previous studies showed that ALK6 was not required for decidualization [12]. In this paper, we demonstrate essential roles of ALK2 in the regulation of endometrial function and female fertility.

## Results

### Generation of *Alk2* cKO mice and *Alk2* expression in the uterus

**Figure 1:**
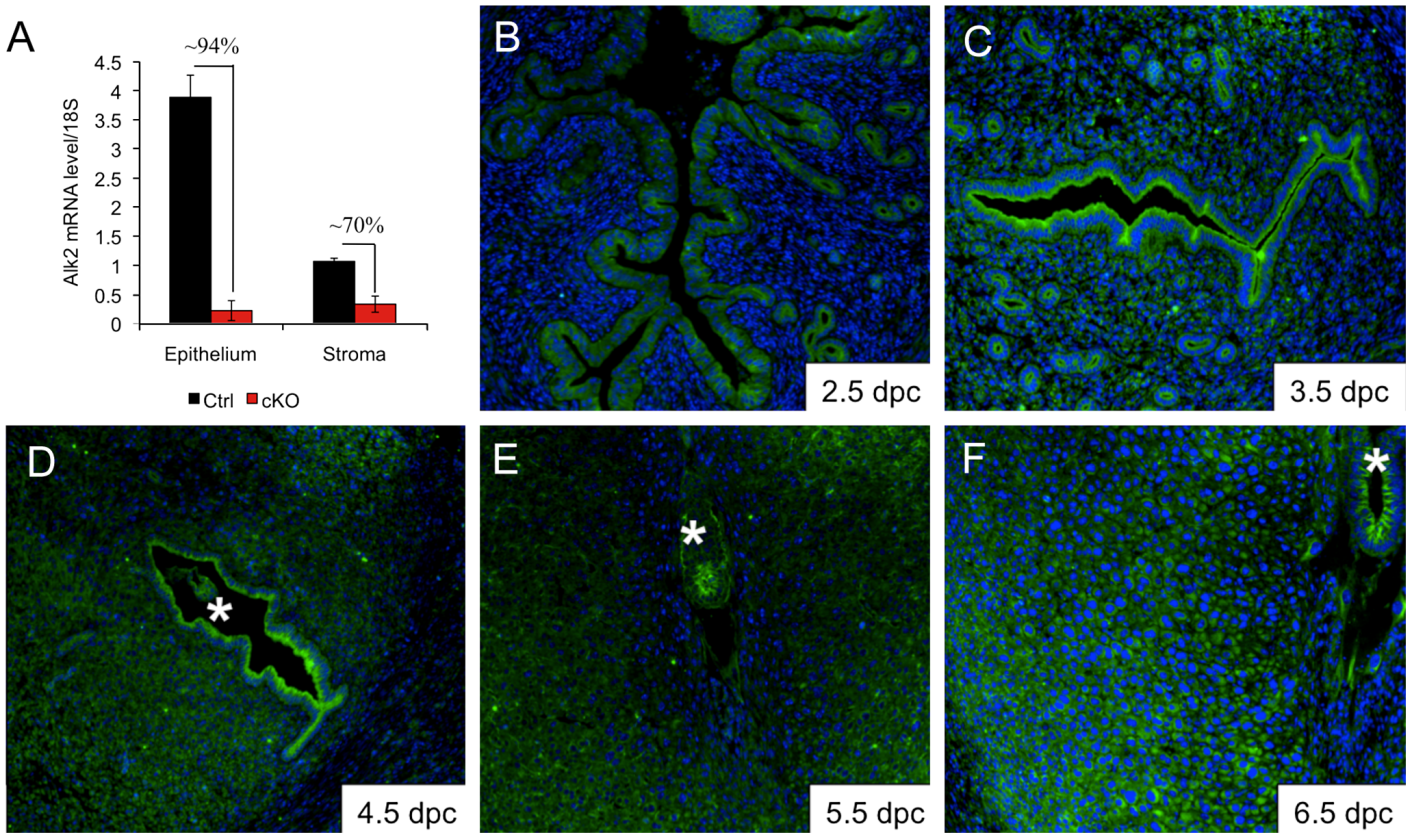
*Alk2* expression in the pregnant uterus. **A**) Relative expression of *Alk2* in the epithelial and stromal compartment of wild type (black columns) and cKO (red columns) mice measured by qPCR. The percentages indicate the reduction of *Alk2* expression due to *cre-*mediated recombination. **B–F**) Immunofluorescence analysis of ALK2 in the mouse uterus during early pregnancy. The embryo is marked with an asterisk.

ALK2 is required during embryonic development, and *Alk2* null mice die at ∼E7.5 because of their inability to complete gastrulation [13], [14]. To study the physiological roles of *Alk2* in female reproduction, we generated a conditional mouse model using *Pgr-Cre* and an allele of *Alk2* in which exon 7 is flanked by *loxP* sites (floxed allele) [15] (Figure S1A). *Pgr-cre* is expressed postnatally in the anterior pituitary, preovulatory granulosa cells of the ovaries, oviduct, and epithelial and stromal compartments of the uterus [6]. Cre*-*mediated recombination was confirmed by performing PCR on uterine DNA collected from *Alk2* control and cKO females; we used primers specific for exon 7 of *Alk2* (Figure S1A) and observed that recombination had efficiently occurred in mice carrying the *Pgr-cre* allele (Figure S1C). To further confirm the efficiency of deletion of *Alk2* in the uterus, we treated ovariectomized mice with 17β-estradiol (E2) and progesterone (P4) to mimic the hormonal levels that naturally occur during blastocyst implantation [16]. We then quantified the expression of *Alk2* by qPCR in samples enriched in the epithelial or stromal component obtained by enzymatic and mechanical separation of the two uterine compartments. *Alk2* mRNA is present in both uterine compartments and the expression in the epithelium is ∼4-fold higher than in the stroma (Figure 1A). In *Alk2* cKO mice, the mRNA level of the receptor was reduced by ∼94% in the epithelium and ∼70% in the stromal compartment (Figure 1A). To further investigate the spatiotemporal localization of ALK2 in the pregnant uterus, we performed immunofluorescence analysis during sequential time points (Figure 1B–F). While ALK2 is not detectable at 2.5 dpc, the receptor is highly expressed on the luminal side of epithelial cells at 3.5 and 4.5 dpc, the period during which the uterine epithelium prepares for and permits the embryo to implant. At these time points, the receptor is also detected in the glandular epithelium but at lower levels in the stromal cells. At 5.5 and 6.5 dpc, ALK2 is highly expressed in stromal cells, and the receptor is more prominently localized in the secondary decidual zone (further from the embryo) than in the primary decidual zone (closer to the embryo). Thus, ALK2 is present in both compartments during pregnancy, and suggests that BMPs play a role both in the epithelium, during the first phases of pregnancy, and in the uterine stroma after implantation.

### *Alk2* cKO females are sterile and show delayed blastocyst implantation and defective decidualization

We next investigated how ablation of ALK2 in the uterus affects female fertility by performing a 6-month breeding trial in which sexually mature control and cKO females (*n = *10 for each group) were mated with wild type males of proven fertility. *Alk2* cKO female mice are sterile, while control littermates display normal fertility and fecundity (9.1±0.2 pups/litter and 1.0±0.2 litter/month), comparable to wild type (8.1±0.3 pups/litter and 1.2±0.1 litter/month) and *Pgr^cre/+^* females (7.8±0.1 pups/litter and 1.1±0.4 litter/month) [17]. The presence of vaginal plugs in *Alk2* cKO females showed that the infertility was not due to abnormal mating behavior. We then performed timed mating experiments in which control and cKO females were mated with wild type males and sacrificed at sequential time points during pregnancy. The attachment of the embryo to the uterine epithelium causes a local increase in vasculature permeability, and implantation sites can be visualized at an early stage by injecting Chicago blue dye intra venous (i.v.). On the day of attachment (i.e., day 4 of pregnancy), we did not observe any difference in the number of sites of attachment between control and cKO females (data not shown). The presence of a normal number of attached embryos shows that the hypothalamic–pituitary–gonadal axis is intact, and ovulation is normal. This suggests that the infertility in *Alk2* cKO females is likely caused by uterine defects.

**Figure 2:**
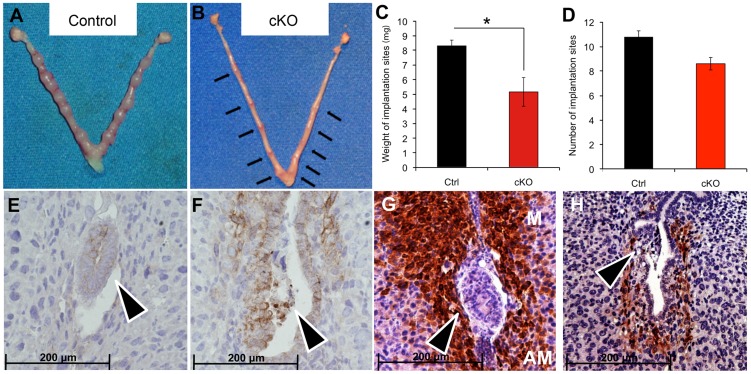
Delayed blastocyst invasion in cKO mice at 5.5 dpc. **A–B**) Gross morphology of the uterus of *Alk2* control (A) and cKO (B) mice. Black arrows indicate the implantation sites in the *Alk2* cKO uterus. **C**) Wet weight of the implantation sites in milligrams (mg.) recovered from pregnant control (black column) and cKO (red column) mice collected at 5.5 dpc. The size of the implantation sites in *Alk2* cKO mice is significantly smaller (*p<0.05). N = 5. **D**) Number of implantation sites in control (black column) and cKO mice (red column). **E–F**) CDH1 (epithelial marker) staining of implantation sites collected from control (E) and cKO (F) mice. Brown color indicates positive staining. Tissue is counterstained with hematoxylin. **G–H**) PTGS2 staining of implantation sites collected from control (G) and cKO (H) mice. Brown color indicates positive PTGS2 signal. M = mesometrial side, AM = antimesometrial side. Arrowhead indicates the embryos. Data are mean ± SEM.

**Figure 3:**
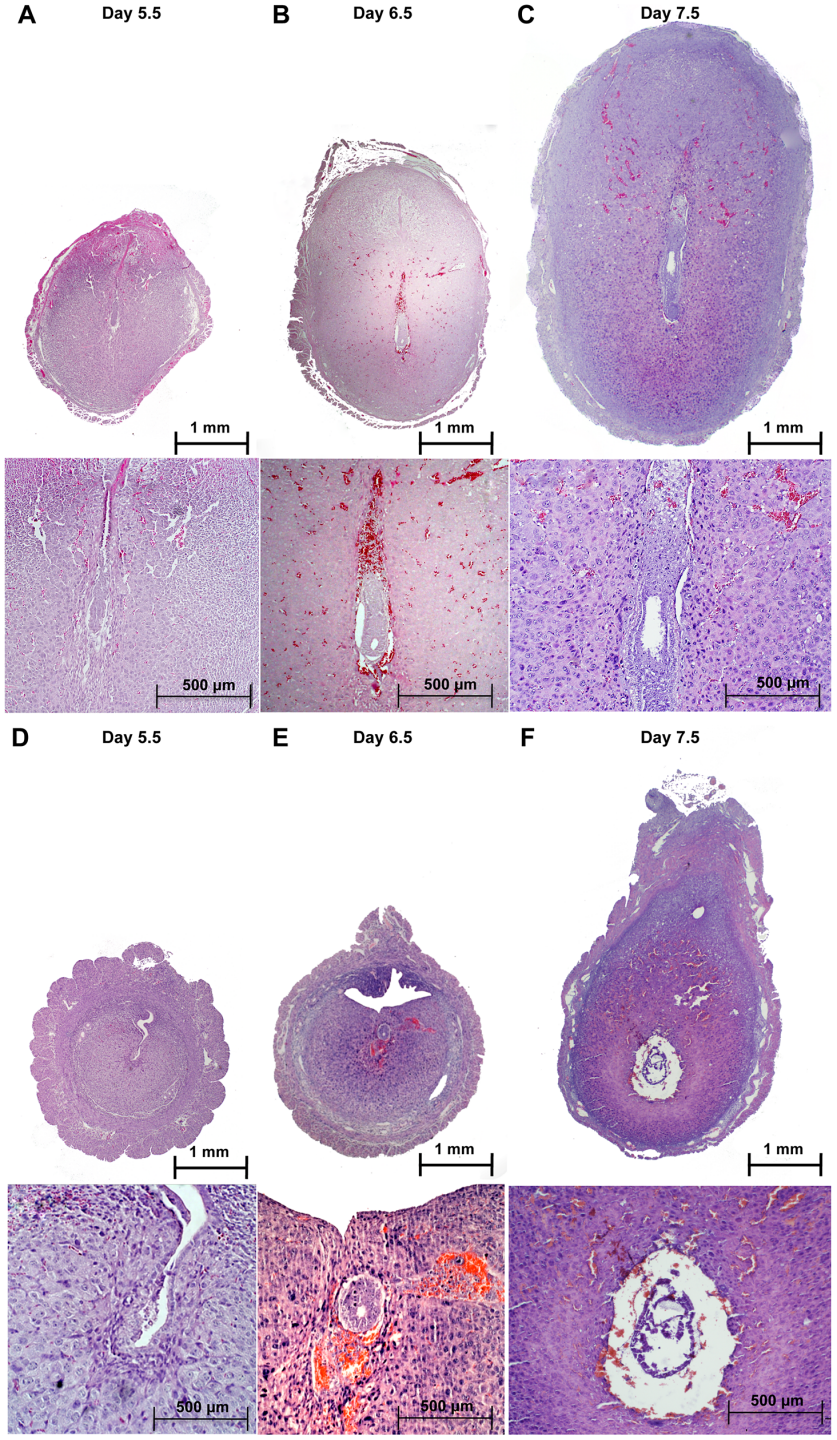
Defective implantation in *Alk2* cKO mice. Implantation sites collected from control (**A–C**) and cKO mice (**D–F**) at sequential time points during pregnancy and stained with H&E.

Upon embryo attachment, the embryo starts invading into the uterine stroma (day 5) and stimulates the differentiation of the stromal cells into decidual tissue, around each implanted embryo. When we inspected the implantation sites at day 5.5 post coitum (5.5 dpc) (Figure 2A–B), we observed that embryos in *Alk2* cKO mice were attached to the luminal epithelium, but the epithelial layer was still intact because the invasion into the stroma had not occurred (Figure 2E–F). Moreover, the uteri of *Alk2* cKO mice still showed similar numbers of implantation sites compared to controls (Figure 2D), but their size and weight were significantly reduced compared to controls (Figure 2C). To further characterize this defect, we collected uteri from pregnant females at 6.5 and 7.5 dpc and examined the histology of the implantation sites (Figure 3). While the embryos eventually invade into the stroma by 6.5 dpc in *Alk2* cKO mice, the process is delayed compared to controls (Figure 3G,L). In addition, embryos that implant into the *Alk2* cKO uteri are consistently smaller compared to controls (Figure 3G–I, L–N) and start to be reabsorbed at 7.5 dpc (Figure 3N). The implantation sites are also smaller in cKO mice compared to controls at all time points observed beginning at 5.5 dpc.

To further study implantation in *Alk2* cKO mice, we analyzed prostaglandin-endoperoxide synthase 2 (PTGS2) protein levels and localization by immunohistochemistry. PTGS2 shows a dynamic localization that precisely correlates with different phases of the implantation process [18]. In particular, PTGS2 localizes to luminal epithelial and subepithelial stromal cells at the antimesometrial pole of the implantation site at the moment of embryo attachment (∼4.0 dpc), and at 5.5 dpc, PTGS2 is produced in decidualizing cells at the mesometrial pole. In control mice, PTGS2 correctly localized at the mesometrial pole at 5.5 dpc. In contrast, in *Alk2* cKO mice, at 5.5 dpc PTGS2 displayed a pattern resembling the moment of attachment (Figure 2G–H), confirming that *Alk2* cKO mice have a delay in the implantation process.

These results show that ALK2 is dispensable for the attachment of the embryo to the uterine epithelium, but is required at a later step during implantation. Ablation of BMP2 has also been shown to disrupt the implantation process soon after the embryo attachment, although the differences in the phenotypes observed between *Alk2* and *Bmp2* cKO mice suggest that BMP2-ALK2 is not the only ligand-receptor pair involved in the regulation of this stage of pregnancy. In fact, the presence of other BMP signaling components in the pregnant mouse uterus suggests that other BMP ligand(s) and receptor(s) can compensate with various degrees of efficiency for the loss of one component of the pathway.

### Ablation of ALK2 compromises uterine decidualization

**Figure 4:**
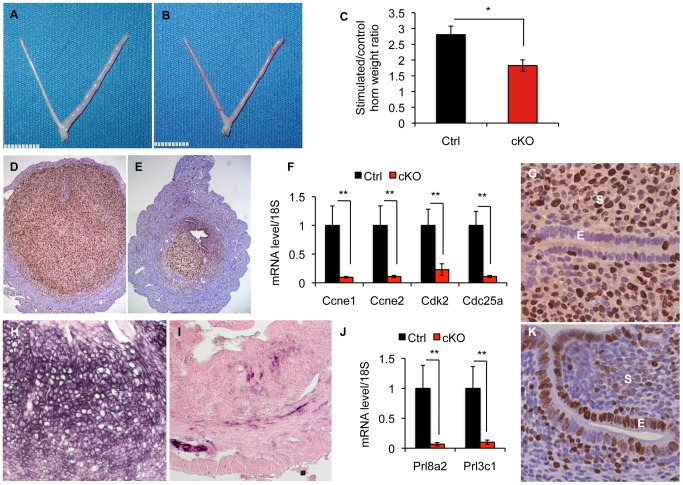
*Alk2* is required during uterine stromal decidualization. Gross morphology of the uteri of *Alk2* control (**A**) and cKO (**B**) mice collected one day after one horn (right) was injected with oil to stimulate the stromal cells to undergo decidualization. The other horn (left) was untreated and used as a control. **C**) Ratio between the wet weight of oil-injected and control horn collected from *Alk2* control (black column) and cKO (red column) mice. The ratio of *Alk2* cKO mice is significantly smaller than in controls (*p<0.05) indicating that a decidualization defect is occurring in the former. N = 5 per genotype. **D–E**) Proliferation of stromal cells during decidualization visualized through Mki67 staining on sections of uteri collected from *Alk2* control (**D**) and cKO (**E**) mice one day after the decidualization was induced. Brown color indicates positive Mki67 staining, thus, proliferating cells. Cell nuclei are stained in blue (hematoxylin). **F**) Expression of some of the main markers of the S phase of the cell cycle (*Ccne1*, *Ccne2*, *Cdk2* and *Cd25*) in the uteri of *Alk2* control (black columns) and cKO (red columns) mice, one day after the decidualization was induced, quantified by qPCR analysis. *Alk2* cKO mice show a significant lower expression of all of these genes compared to controls (** p<0.01). N = 5. **G,K**) Epithelial proliferation in *Alk2* cKO mice. Sections of uterine tissues obtained from implantation sites of control (**G**) and cKO mice (**K**) were stained for the proliferation marker Mki67. A striking increase in the amount of proliferating cells was observed in cKO mice compared to controls. E = epithelium, S = stroma. **H–I**) Differentiation of stromal cells during decidualization visualized through the evaluation of endogenous alkaline phosphatase (ALP) activity. Black color indicates positive ALP staining. **J**) Expression of differentiation markers (*Prl8a2* and *Prl3c1*) in decidualizing uteri of *Alk2* control (black) and cKO (red) mice, evaluated by qPCR. *Alk2* cKO mice show a significant lower expression of both genes (** p<0.01). N = 5. Data are means ± SEM.

Invasion of an embryo into the uterine wall stimulates stromal cells to undergo a functional and morphological transformation called decidualization. During this process, stromal cells of the uterus extensively proliferate and differentiate to create a permissive environment for the implanting embryo. Because *Alk2* cKO mice show smaller implantation sites, we decided to further investigate how ablation of ALK2 affects the proliferation and differentiation of the uterus when a decidualizing stimulus is given. The uterus is receptive only for a limited period of time during which the sex hormones prepare it to adequately respond to the attachment of the embryo. Because sex hormones are produced by the ovaries in cyclic waves, we ovariectomized and injected mice with sex hormones to artificially induce receptivity and exogenously control and synchronize the cycle of the mice in our experiments. We mimicked the implantation of the embryo by injecting one horn with oil. Consistent with the reduced size of implantation sites during natural pregnancy, we observed that *Alk2* cKO females undergo a significantly reduced increase in uterine horn weight one day after oil injection compared to control littermates (Figure 4A–C). At five days, the difference in size of the stimulated to untreated horn of *Alk2* cKO mice to control mice was even greater (Figure S2A–C). These results confirm that the uteri of cKO mice are not able to decidualize in response to the attachment of the embryo.

We next investigated the ability of stromal cells to proliferate and differentiate in response to the decidual stimulus. Both control and cKO mice show extensive stromal proliferation in the uterus (detected by MKi67 staining), but the amount of cells in the stromal compartment of cKO mice was reduced compared to controls, suggesting an impairment of the process (Figure 4D–E). Moreover, cKO mice showed a significantly reduced expression of several molecular markers of the S phase of the cell cycle (*Ccne1*, *Ccne2*, *Cdk2*, and *Cdc25*) (Figure 4F). To investigate whether the differentiation of stromal cells is also impaired by the ablation of ALK2, we evaluated alkaline phosphatase activity (ALP) in stromal cells one day after decidualization was induced. While control mice showed a strong purple signal in the uterine stromal compartment (indicating the presence of alkaline phosphatase activity, and thus differentiation), we observed a lack of differentiating cells in the uteri of cKO females (Figure 4I–J). In addition, qPCR analysis of the two differentiation markers *Prl8a2* and *Prl3c1* confirmed a significant impairment of differentiation at a molecular level (Figure 4K). These data demonstrate that signaling through ALK2 is required during decidualization to achieve stromal cell proliferation and differentiation.

Although stromal cell proliferation and differentiation was reduced, Ki67 staining also revealed a pronounced proliferative activity in the epithelium of cKO mice that is not present in control mice (Figure 4G–H). E2 stimulates the proliferation of uterine epithelial cells in preparation for implantation, and this proliferative action is inhibited by P4 acting through its receptor, PGR [16]. These observations led us to hypothesize that ALK2 acts to regulate stromal cell proliferation and repress epithelial cell proliferation by regulating the expression and/or activity of PGR. However, when we assayed the levels of PGR in the uterus one day after the induction of decidualization, we did not observe any difference in the total amount nor cellular localization of PGR protein in control and cKO mice, with PGR mainly localized in the nuclei of stromal cells (Figure S4A–D).

### Microarray analysis of *Alk2* cKO uteri indicates that *Cebpb* is regulated by BMPs during decidualization

**Figure 5:**
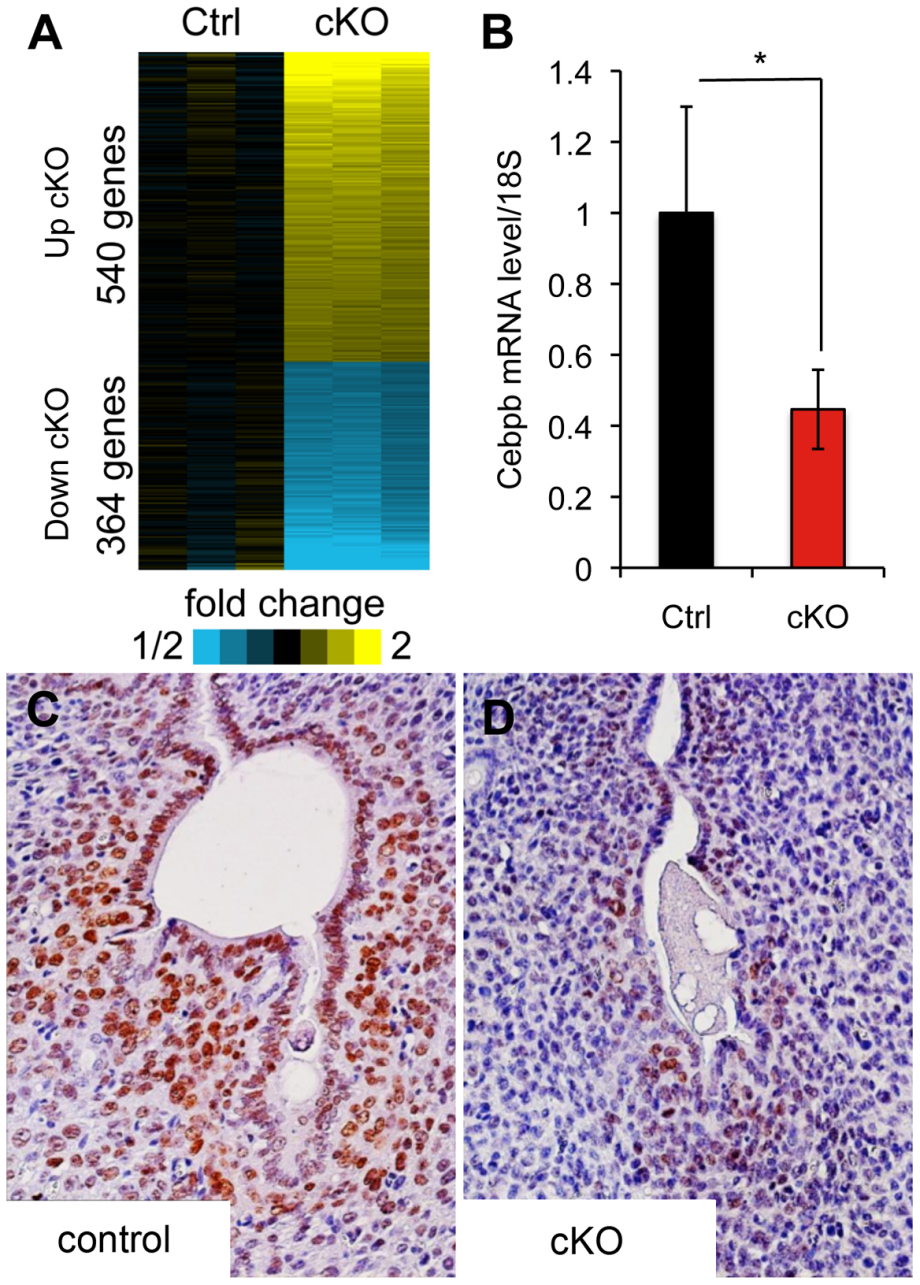
Microarray analysis of *Alk2* control and cKO uteri at day one of decidualization. **A**) Heat map of genes upregulated (yellow) and downregulated (blue) in *Alk2* cKO (right) mice compared to controls (left). Each column represents the expression data obtained from a pool of RNA collected from 3 mice with the same genotype. Three samples of pooled RNA per each genotype were submitted for microarray analysis. **B**) Expression of *Cebpb* in the uteri of *Alk2* control (black column) and cKO (redcolumn) mice collected one day after the induction of decidualization, quantified by qPCR analysis. *Alk2* cKO mice show a significantly lower expression of *Cebpb* compared to controls (* p<0.05). N = 5. **C–D**) CEBPB staining on sections of *Alk2* control (**C**) and cKO (**D**) decidualized uteri collected one day after oil injection. Brown color indicates positive CEBPB staining. Cell nuclei are stained in blue (hematoxylin). Data are means ± SEM.

To investigate the downstream pathways controlled by BMPs during the decidualization process, we performed microarray analysis on control and cKO uteri collected one day after artificial induction of decidualization. We found that the expression of 909 unique genes is significantly affected by the ablation of ALK2 (p<0.01, fold change <0.7, >1.3) (Figure 5A). We used the **D**atabase for **A**nnotation, **V**isualization and **I**ntegrated **D**iscovery (**DAVID**) to identify the main pathways controlled by BMPs during decidualization. During decidualization, stromal cells undergo a wave of proliferation and differentiation that lead to the formation of a specialized tissue (the decidua) able to sustain the growth of the implanted embryo. Our data show that without ALK2, stromal cells cannot respond to a decidual stimulus by normally proliferating and differentiating. Thus, the significant alteration in the nucleotide synthesis pathways and DNA metabolic process that we observe in *Alk2* cKO mice is likely a consequence of the defect in proliferation; moreover, because there is a profound remodeling of the vasculature during decidualization that is tightly regulated by immune system factors, it was not surprising to see an effect of ALK2 ablation on pathways involved in vasculature development and immune response (Table 1).

**Table 1:** Microarray data analysis, GO terms with p<0.025.

Term	Count	P-value
Nucleotide biosynthetic process	18	9.60E-05
Nitrogen compound biosynthetic process	29	2.50E-06
Steroid metabolic process	15	1.90E-03
Response to endogenous stimulus	16	2.60E-03
Locomotory behavior	19	1.80E-03
Behavior	31	5.70E-05
Skeletal system development	21	2.70E-03
Cellular macromolecular complex assembly	15	1.30E-02
Membrane organization	20	4.20E-03
Enzyme linked receptor protein signaling pathway	20	4.20E-03
Leukocyte activation	15	2.00E-02
Cellular macromolecular complex subunit organization	16	1.90E-02
Blood vessel development	17	1.70E-02
Cell activation	16	2.50E-02
In utero embryonic development	17	2.10E-02
Macromolecular complex assembly	21	1.10E-02
Immune response	28	6.70E-03
Chordate embryonic development	25	1.30E-02
DNA metabolic process	24	1.70E-02
Oxidation reduction	39	2.30E-03

Another Gene Ontology (GO) term that we found to be significantly affected in the array analysis comprises genes involved in the regulation of *in utero* development (Table 2). Among these genes is CCAAT/enhancer-binding protein beta (*Cebpb*), which has been shown to regulate the proliferation and differentiation of stromal cells during decidualization. Similar to *Alk2* cKO females, mice with a null mutation in *Cebpb* are not able to decidualize [19]. We used qPCR and immunohistochemistry to confirm that the expression of *Cebpb* mRNA and CEBPB protein levels are significantly reduced in *Alk2* cKO mice compared to controls during decidualization (Figure 5B–D). Although both ALK2 and CEBPB are required for stromal cell proliferation during decidualization, the two factors seem to regulate different phases of the cell cycle; while *Cebpb* cKO mice show a disruption of expression of factors controlling the G2 to M phase transition of the cell cycle, *Alk2* cKO mice display an alteration at the S phase. These data suggest that BMP signaling acts upstream to regulate the expression of the transcription factor *Cebpb*, and this regulatory mechanism is required for the progression of the cell cycle in uterine stromal cells during decidualization.

**Table 2:** Genes involved in the regulation of *in utero* development.

Official gene symbol	Gene name	Fold change
**BCL2L11**	BCL2-like 11 (apoptosis facilitator)	1.53
**CEBPB**	CCAAT/enhancer binding protein (C/EBP), beta	0.69
**NSDHL**	NAD(P) dependent steroid dehydrogenase-like	0.65
**AMD2**	S-adenosylmethionine decarboxylase, pseudogene 7; S-adenosylmethionine decarboxylase, pseudogene 6; S-adenosylmethionine decarboxylase, pseudogene 4; S-adenosylmethionine decarboxylase 1; S-adenosylmethionine decarboxylase, pseudogene 3; similar to S-adenosylmethionine decarboxylase proenzyme 2 (AdoMetDC 2) (SamDC 2)	0.64
**TEAD4**	TEA domain family member 4	0.58
**EPAS1**	Endothelial PAS domain protein 1; similar to endothelial PAS domain protein 1	0.58
**EDNRA**	Endothelin receptor type A	1.54
**GJA1**	Gap junction protein, alpha 1	0.68
**GAB1**	Growth factor receptor bound protein 2-associated protein 1	1.47
**HAND2**	Heart and neural crest derivatives expressed transcript 2	0.63
**LEF1**	Lymphoid enhancer binding factor 1	0.6
**NOG**	Noggin	1.84
**NASP**	Nuclear autoantigenic sperm protein (histone-binding); similar to nuclear autoantigenic sperm protein; NASP	0.61
**PDGFRB**	Platelet derived growth factor receptor, beta polypeptide	1.46
**PSMC4**	Proteasome (prosome, macropain) 26S subunit, ATPase, 4	0.7
**SERPINA1B**	Serine (or cysteine) peptidase inhibitor, clade A, member 1B	2.43
**SLC34A2**	Solute carrier family 34 (sodium phosphate), member 2	1.48

### ALK2 is required for decidualization in human endometrial stromal cells (hESC) and controls the expression of *CEBPB* and *PGR*

**Figure 6:**
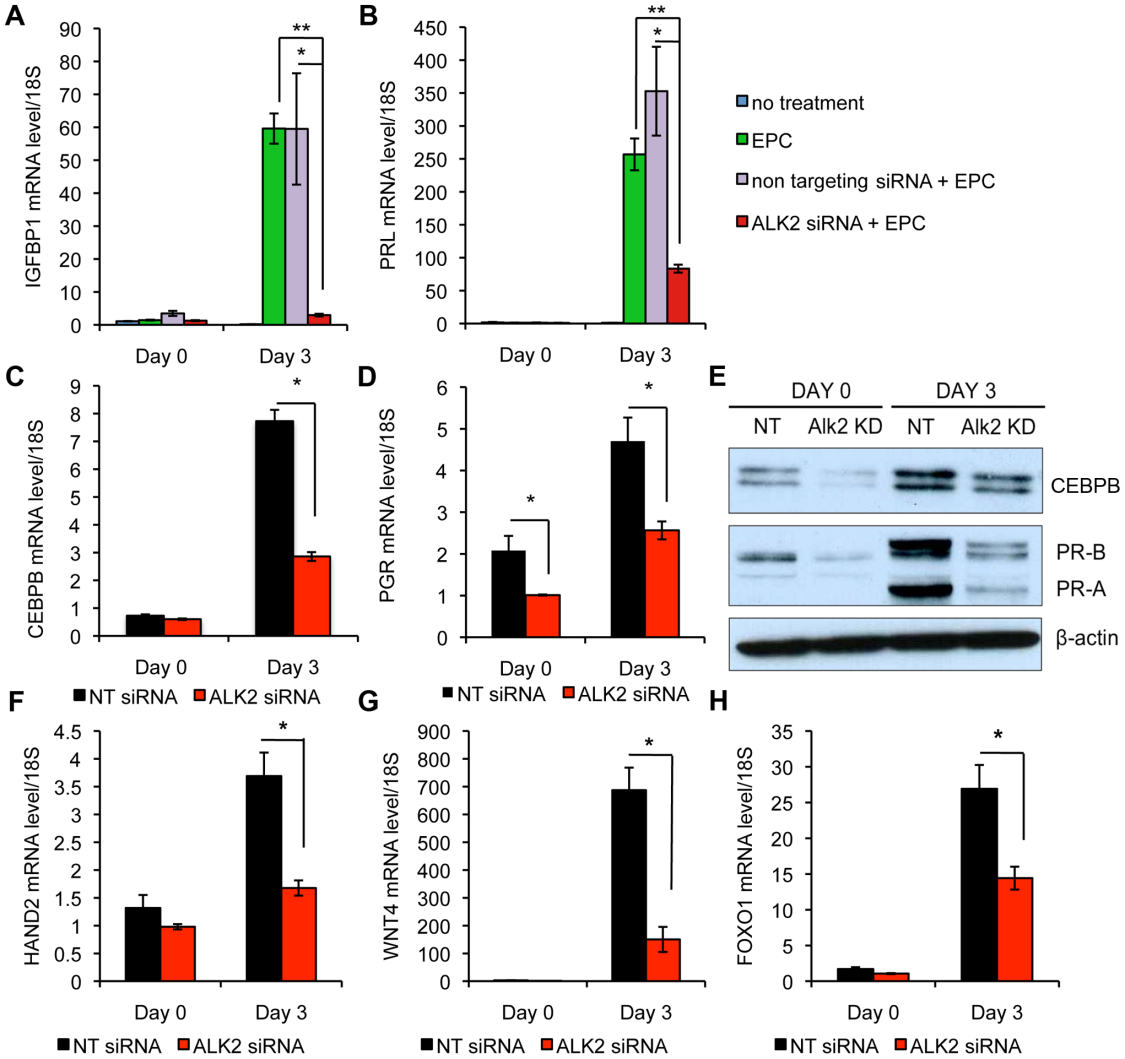
*Alk2* is required during the decidualization of human endometrial stromal cells. Expression of decidual markers, *IGFBP1* (**A**) and *PRL* (**B**) in human endometrial stromal cells (hESC) cultured in growth medium (blue columns), treated with a hormonal cocktail containing estradiol, medroxyprogesterone acetate (MPA) and cAMP (EPC, green columns), treated with EPC after transfection of non targeting siRNA (red columns) or siRNA targeted to *ALK2* (purple columns). Day 0 and day 3 refer to EPC treatment; the siRNA transfections were performed at day −1 and −2. (* p<0.05, ** p<0.01). N = 3. **C–D**) Expression of *CEBPB* (**C**) and *PGR* (**D**) at day 0 and after 3 days of EPC treatment in cells treated with non targeting (NT) siRNA (black column) and siRNA targeted to *ALK2* (*ALK2* siRNA, red column) (* p<0.05). N = 3. **E**) CEBPB and PGR (isoform A and B) protein levels in NT siRNA-treated and *ALK2* siRNA-treated hESC, quantified by western blotting at day 0 and day 3 of EPC treatment. β-ACTIN was used as loading control. **F–H**) Expression of *HAND2* (**F**), *WNT4* (**G**) and *FOXO1* (**H**) at day 0 and day 3 of EPC treatment in NT siRNA-treated (black column) and *ALK2* siRNA-treated (red column) hESC (* p<0.05). N = 3. Experiments were performed on cells from 3 patients. Data are means ± SEM.

In many species, endometrial stromal cell decidualization is induced upon implantation of the embryo. In humans, this process takes place during the mid-secretory phase of each menstrual cycle, independently of pregnancy. Similar to the mouse, decidualization in humans is a process of dramatic transformation of endometrial stromal fibroblasts into secretory, epithelioid-like decidual cells. To study decidualization in humans, this process can be recapitulated in primary cultures of human endometrial stromal cells (hESC) [20]. Treatment of hESC with a cyclic AMP analog triggers the expression of decidual marker genes like prolactin (*PRL)* and insulin-like growth factor binding protein 1 (*IGFBP1)* within hours and, via activation of protein kinase A (PKA), sensitizes hESC to progesterone action. When hESC are treated with both cAMP and P4, the expression of decidual markers is enhanced and the decidual phenotype is maintained for a longer period. We utilized hESC to investigate the role of ALK2 during decidualization in human. hESC were first treated with either non-targeting (NT) small interfering RNA (siRNA) or siRNA targeted to *ALK2* (*ALK2* knock down, KD), then stimulated to decidualize with a hormonal cocktail containing estradiol, medroxyprogesterone acetate (MPA) and cAMP (EPC). After 3 days of treatment, we used qPCR to measure the expression of *IGFBP1* and *PRL*. Both decidualization markers show a significant reduction in *ALK2* KD cells compared to NT-treated cells (Figure 6A–B), indicating that ALK2 is required during decidualization both in mouse and human. Upon differentiation of the stromal compartment, many genes first expressed in the epithelium, are induced in decidual cells. CEBPB has been shown to be required for human and murine decidualization and the physical interaction between CEBPB and PGR has been demonstrated using an *in vitro* system [21]–[23]. Because we found *Cebpb* to be downregulated during decidualization in the mouse, we were interested in testing whether BMPs control the expression of *CEBPB* in human endometrial stromal cells as well. Both *CEBPB* mRNA and protein level were significantly lower in *ALK2* KD cells compared to NT-treated cells, indicating that the involvement of the BMP-CEBPB pathway in the regulation of decidualization is conserved in mice and humans (Figure 6C,E).

Progesterone acts through its cognate receptor, the nuclear progesterone receptor (PGR), to regulate implantation and decidualization via the activation of several downstream pathways [24]. Although *Alk2* cKO mice show a desensitization to progesterone, neither the PGR mRNA nor protein levels show a significant alteration in the cKO mice. It has been suggested that BMPs are required for the expression of FK-506-binding proteins (FKBPs) that, in turn, control the activity of PGR by binding and maintaining the receptor in a functional state. This hypothesis is confirmed by the observation that *Fkbp4* and *Fkbp5* are downregulated in *Alk2* cKO mice during decidualization (Figure S3A–B). To investigate whether the same mechanism was involved in the control of decidualization in hESC, we first measured the expression and translation of *PGR* in NT-treated and *ALK2* KD cells. In contrast to the mouse, we found that ablation of ALK2 in human cells causes a downregulation of progesterone receptor at both the mRNA and protein levels. Because the levels of PGR are reduced in *ALK2* KD cells even before decidualization was induced (day 0), the downregulation of PGR is not a consequence of the lack of decidualization but is a direct effect of the ablation of ALK2. To confirm the hypothesis that ALK2 is required for functional PGR activity, we measured the expression of known PGR targets that are normally activated by PGR during decidualization. The expression of all the PGR targets that we quantified by qPCR [i.e., wingless-type MMTV integration site family, member 4 (*WNT4*), forkhead box O1 (*FOXO1*), and heart and neural crest derivatives expressed transcript 2 (*HAND2*)] is significantly reduced in *ALK2* KD cells (Figure 6F–H). These findings show that ALK2 is required during the decidualization of hESC, and the mechanism through which it acts likely involves the regulation of *CEBPB and PGR*. The regulation of *CEBPB*, but not PGR levels, by BMPs during decidualization, is conserved between mice and humans.

### Chromatin immunoprecipitation (ChIP) analysis reveals that BMPs directly regulate *CEBPB*, and CEBPB activates *PGR* in decidualizing hESC

**Figure 7:**
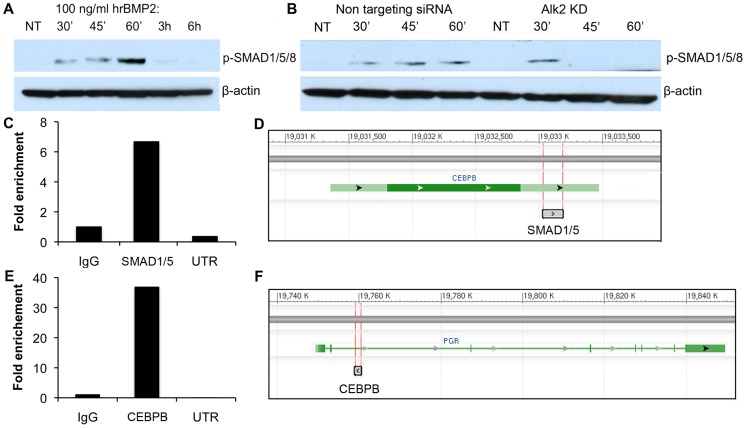
SMAD1,5,8 phosphorylation in hrBMP2-treated human endometrial stromal cells and SMAD1/5 and CEBPB ChIP experiments. **A**) Phosphorylation of SMAD1,5,8 in hESC treated with 100 ng/ml hrBMP2 for 0 (non treated = NT), 30, 45, 60 minutes, 3 or 6 hours. B-ACTIN was used as loading control. **B**) Phosphorylation of SMAD1,5,8 in hESC transfected with NT (left) or *ALK2* siRNA (right) and treated with 100 ng/ml hrBMP2 for 0 (NT), 30, 45 or 60 minutes. **C**) qPCR quantification (Sybr green) of DNA recovered after pull down with SMAD1/5 or CEBPB (**E**) antibody. We used a pair of primers (Table S1) specific for putative binding sites in proximity of the *CEBPB* (**D**) and *PGR* (**F**) genes. The quantification is given as fold enrichment of SMAD1/5- or CEBPB-pulled down vs. IgG-pulled down DNA. UTR = untranslated region used as negative control (primers targeting a fragment from a gene desert on chromosome 4).

BMPs exert their action on target cells by causing the phosphorylation and activation of their intracellular effectors, SMAD1, SMAD5 and SMAD8. These proteins, once activated, bind SMAD4 and accumulate in the nucleus where they activate the expression of a variety of BMP target genes. To test whether the canonical BMP-SMAD pathway was active in hESC, we treated the cells with recombinant human BMP2 (rhBMP2) and measured the phosphorylation of SMAD1, 5 and 8 at sequential time points. The decision to use rhBMP2 was driven by the known central role of this factor during decidualization in both mouse and human [8], [9]. As expected, we observed that rhBMP2 causes the phosphorylation of SMAD1, 5 and 8 between 30 minutes and 1 hour of treatment (Figure 7A). We then repeated the same experiment using hESC treated with either NT-siRNA or *ALK2-siRNA*. Because *ALK2* KD cells displayed a strong decrease in SMAD1, 5, 8 phosphorylation (Figure 7B), we propose that BMP2 signals through ALK2 in hESC in a SMAD1, 5, 8-dependent manner to regulate the stromal decidualization. Nevertheless, some residual phosphorylation can still be observed at 30 minutes in *ALK2* KD cells, and we suggest that this is due to a redundant action of another BMP type 1 receptor.

Because progesterone has been shown to regulate the expression of *CEBPB* in the uterus, we tested whether BMPs directly regulate *CEBPB* expression or if the downregulation of *CEBPB* in *ALK2* KD cells is secondary to an alteration in *PGR* expression and/or activity. To test these possibilities, we performed ChIP analysis of SMAD1/5 on putative DNA binding regions in the proximity of the *CEBPB* gene in hESC induced to decidualize. We found a significant enrichment of one of the putative regions in the DNA immunoprecipitated using an antibody directed against SMAD1/5, indicating the presence of SMAD1/5 on the *CEBPB* 3′ UTR during decidualization (Figure 7C–D). These findings confirm that BMPs regulate the expression of *CEBPB* during hESC decidualization and show that this regulation occurs directly through binding of SMAD1/5 to the 3′UTR of *CEBPB*.

Because we also observed a decrease of PGR in *ALK2* KD hESC, and CEBPB has been shown to regulate the expression of *PGR* in the mammary gland during alveologenesis (unpublished), we wanted to test whether CEBPB regulates the expression of *PGR* in hESC during decidualization. We performed ChIP analysis of CEBPB on putative binding sites in the proximity of *PGR* and found a 35-fold enrichment of one of the four sites that we tested (Figure 7E–F and Table S1). Our results show for the first time a direct regulatory connection between BMPs, CEBPB, and PGR, three of the main regulators of decidualization.

## Discussion

The data presented in this paper show evidence that BMPs play a central role during early pregnancy in both human and mouse. In particular, we discovered that the BMP type 1 receptor ALK2 is required in the uterus for normal embryo implantation and subsequent decidualization of stromal cells. To implant, the embryo first attaches to the uterine epithelium, then invades into the uterine stroma. The uterine stroma responds to the embryo by undergoing stromal cell decidualization. Without uterine ALK2, embryos start invading into the stroma one day later than in control mice, show growth retardation, and die by 7.5 dpc. In contrast, the ablation of BMP2 in the uterus causes the arrest of pregnancy at the attachment phase [8]. We suggest that without ALK2, other receptors partially compensate and mediate BMP2 signaling to allow the completion of implantation.

Both *Bmp2* and *Alk2* cKO mice show a defect in stromal cell proliferation and differentiation during decidualization. By using microarray expression analysis, we investigated the molecular pathways affected by the ablation of ALK2 during the induction of decidualization. It was not surprising to see that *Bmp2* cKO and *Alk2* cKO mice show a significant number of common downstream targets. In particular, in both mouse models there is a downregulation of the immunophilins, *Fkbp4* and *Fkbp5*. These FKBP proteins can associate with heat shock proteins (HSP70 and HSP90) and regulate the intracellular trafficking and function of steroid hormone receptors [25]. Because the expression of *Fkbp4* and *Fkbp5* is repressed in the absence of BMP2 or ALK2, the function of PGR is impaired and decidualization is defective. The impairment of PGR activity caused by the ablation of ALK2 also causes a perturbation of the uterine epithelium, It is known that the proliferation of uterine epithelial cells is controlled by sex hormones: during each estrous cycle, in preparation for implantation, an increase in E2 levels causes a wave of cell proliferation; this is followed by a surge of P4 that inhibits the E2-dependent proliferation and stimulates cells to differentiate [26]. *Alk2* cKO mice show retention in epithelial proliferation when decidualization is artificially induced and during natural pregnancy, suggesting that the absence of ALK2 causes desensitization to progesterone.

In preparation for embryo attachment, PGR activates the expression of heart and neural crest derivatives expressed 2 (*Hand2*) in the stroma, and this, in turn, represses the FGF pathway that acts in a paracrine fashion to inhibit the ER-mediated proliferation in the epithelium [27]. Our results show that *Alk2* cKO mice display retention in epithelial proliferation without showing a significant change in the mRNA or protein levels of HAND2 (Figure S7C–E). ALK2 can regulate the epithelial proliferation during pregnancy through two alternative mechanisms. First, ALK2 may regulate the inhibitory activity of stromal PGR through a HAND2 independent mechanism. Second, ALK2 may repress epithelial cell proliferation directly. It has already been demonstrate that epithelial PGR can directly regulate the expression of epithelial target genes [28], and signaling through ALK2 in the epithelium may mediate this repression. When we analyzed the level of PGR in the uterus during decidualization, we could not detect any significant difference in the amount or histological localization of the protein in the uterine epithelium and stroma (Figure S4A–D). This observation confirms that PGR activity, rather than its expression or translation, is affected by the ablation of ALK2 in the mouse.

ALK2 is localized in both epithelium and stroma during pregnancy, and its ablation affects both compartments: embryos show delayed implantation, and the stromal cells of the uterus do not respond to the embryo by normally decidualizing. Based on these results, it is not possible to establish whether the two defects are caused by the impairment of the same mechanism or if ALK2 controls two independent processes in the two compartments. Because we investigated decidualization by using an artificial induction experiment, we can conclude that the decidualization defect is not due to the delayed invasion of the embryo. However, we cannot determine whether decidualization is affected by a lack of ALK2 in the epithelium or in the stroma. In fact, both paracrine and autocrine factors seem to be involved in regulating this process. Because the uterus requires an intact epithelium to decidualize [29], it has been suggested that some signal from the epithelium is required to have a functional response in stromal cells. It is possible that the lack of ALK2 in the epithelium makes stromal cells unresponsive to the invasion of the embryo, likely through a deregulation of either epithelial or stromal progesterone receptor activity. A role of ALK2 in epithelial cells in the mouse is supported by the observation that, ablation of ALK2 only in the stromal compartment by using the *Amhr2^cre/+^* mouse model, does not significantly affect the fertility of the mice (Figure S8A–B). However, the efficiency of the KO and the residual presence of ALK2 in this model have not been investigated and the normal fertility could be explained by a partial retention of ALK2.

When siRNA targeted to *ALK2* is used to silence the receptor in human endometrial stromal (hES) cells, the expression of the decidual markers *IGFBP1* and *PRL* is significantly repressed compared to hES cells treated with non-targeting siRNA. These results suggest that ALK2 is required in the human uterine stroma during decidualization. Although this *in vitro* model of decidualization has been largely used by others [30] and is commonly accepted as a valid tool to recapitulate the decidualization process in cultured cells, the limitations of this simplified, artificial system must still be taken into account before drawing conclusions. However, the similarities of the findings made by using a combination of *in vitro* and *in vivo* systems strongly suggests a certain level of conservation. For instance, in both the mouse and in human cells, BMP2 and ALK2 have been shown to be required for implantation and decidualization and the pathways downstream to BMP2 in decidualization have been shown to be conserved in mouse and human cells [9]. Furthermore, in both mouse and human cells, CEBPB levels are decreased without ALK2, suggesting that the same downstream pathways are affected.

Our data show that when ALK2 is ablated in hES cells, several pathways are affected. In particular, we saw that the presence of ALK2 is required for the expression of two important regulators of decidualization: *CEBPB* and *PGR*. Although the role of these factors in decidualization has been already demonstrated, we found that BMP, CEBPB and PGR signaling are directly connected and can regulate each other's expression during decidualization. P4 activates *Bmp2* and *Cebpb* expression in the mouse uterus [4], [19] and, in hES cells, we show that BMPs signal through ALK2 and SMAD1/5 to promote *CEBPB* expression. Finally, CEBPB binds to intron 2 of *PGR*. In the mouse, we also show that BMP signaling is required for the expression of *Cebpb*, but the expression of *Pgr* is not reduced in mice lacking uterine ALK2. The results we obtained in hESC suggest a mechanistic solution to the phenotype that we observe in the *Alk2* cKO mouse. In total, these data support a fundamental role of ALK2 in both species.

In humans, infertility is one of the most common reproductive disorders, with 10–15% of couples finding it difficult or impossible to conceive [3]. The implantation of the embryo in the uterus is one of the most critical steps of pregnancy. Approximately 75% of pregnancy failures are due to implantation defects [31]–[33]. The implantation rates in the assisted reproductive technology (ART) laboratory rapidly decrease with the age of the female patient, going from 30.8% in women under 35 year old (y.o.) to 8.2% in the upper age range (41–42 y.o.) (SART 2009). Furthermore, early pregnancy loss occurs in up to ∼50% of patients [34], [35]. Even when defective implantation does not cause pregnancy loss, it can still be the cause of several later complications [i.e., spontaneous abortion, preeclampsia (high blood pressure and protein in the urine), preterm labor, premature rupture of the membranes, intrauterine growth retardation, and disorders of placental attachment] [32], [35]. Roles of BMP2 and activin A in the process of decidualization has been shown *in vitro* using human endometrial stromal cells and *in vivo* for BMP2 using a mouse model [8], [9], [36]. We now show that ALK2 is another signaling component of the TGFβ superfamily involved in the regulation of implantation and decidualization in both mouse and human. Because the ablation in the uterus of BMP2 compared to its receptor ALK2 affects implantation of the embryo at different stages, we suggest that other BMP ligands and/or receptors are also involved in the regulation of early stages of pregnancy.

In our current study, we further clarify the molecular mechanism through which BMPs act in the uterus during decidualization, and we demonstrate for the first time a direct connection between BMP, CEBPB, and PGR pathways. A better knowledge of the molecular mechanisms involved in the regulation of implantation will be critical for developing drugs designed for improving pregnancy rates or, alternatively, for developing new forms of contraceptives that avoid the side effects of the current hormonal contraceptives, such as an increased risk of blood clots, high blood pressure, and breast cancer [37]–[39]. Furthermore, the involvement of TGFβ signaling in a broad array of developmental and physiological processes [40] makes the investigation of the pathway a topic that goes well beyond the scope of the field of reproductive biology. New drugs acting on the TGFβ pathway could be suitable for use in the treatment of a variety of human diseases ranging from cancer, fibrosis and cardiovascular diseases to metabolic disorders like diabetes and obesity [41]–[47].

## Materials and Methods

### Generation of *Alk2* conditional knockout female mice

Mice carrying the *Alk2* conditional allele (*Alk2^flox/flox^*) were created by flanking *Alk2* exon 7, encoding a critical portion of the kinase domain, with two *loxP* sites [15]. The *Alk2^flox/flox^* mice were bred to mice carrying progesterone receptor-*cre* knock-in (*Pgr^cre/^* ^+^) allele [6] to generate *Alk2^flox/flox^Pgr^cre/+^* mice, which were designated as *Alk2* cKO. *Alk2^flox/flox^* female mice were used as controls. Mice were genotyped by PCR analyses of genomic tail DNA as described by others [48]. Analysis of DNA recombination in the uterus was performed using DNA isolated from uterine tissue. All mouse lines used in the present study were maintained on a hybrid C57BL/6J and 129S7/SvEvBrd genetic background. Animal handling and surgeries were performed according to the NIH *Guide for the Care and Use of Laboratory Animals*. All procedures have been approved by the Institutional Animal Care and Use Committee (IACUC) at Baylor College of Medicine.

### Hormone treatments

Physiological concentrations of E2 and P4 were given exogenously to mice to recapitulate the hormonal levels occurring during early pregnancy as previously described [16]. Briefly, 6–8 week old mice were ovariectomized via a dorsal incision under 2,2-tribromoethanol (2.5% Avertin) anesthesia and left untreated for 2 weeks to achieve complete withdrawal of endogenous ovarian hormones; then, mice were “primed” with daily subcutaneous (s.c.) injections of 100 ng of 17β-estradiol (E2) for 2 days. After 2 days of rest, mice were injected for 3 days with 1 mg of progesterone (P4) daily and on the fourth day with 1 mg of P4 and 50 ng of E2. Mice were sacrificed 6 hours after the last injection.

The artificial induction of decidualization has been previously described [26]. Briefly, control and cKO mice were ovariectomized and, after 2 weeks, treated with s.c. injections of 100 ng of E2 once a day for 3 days. After 2 days rest, mice were injected daily with P4 (1 mg, s.c.) and E2 (6.7 ng, s.c.) once a day for 3 days. One uterine horn was then treated with a decidual stimulus by injecting 200 µl of sesame oil into the lumen 6 hour after the last hormone injection. The contralateral horn was not injected and served as a control. The next day, mice were given a final injection of P4 (1 mg, s.c.) and E2 (6.7 ng, s.c.) and euthanized 6 hours later; wet weight of the traumatized and control horns was recorded. Another group of mice was injected for 5 days after the oil injection and sacrificed 6 hours after the last injection. Upon euthanasia, the uterine tissue was collected and either fixed in 4% paraformaldehyde (PFA) or frozen for RNA extraction.

### Epithelial-stromal separation

To separate the epithelial and stromal components of the uterine tissue, uteri were collected, cut in 1–2 mm pieces and incubated in Hank's balanced salt solution (HBSS, Invitrogen) +1% trypsin (Sigma) for 3–5 hour at room temperature. Afterwards, tissues were washed in PBS and luminal epithelium was mechanically separated from the stromal compartment under a dissecting microscope using dissecting forceps and a mouth pipette. To ensure adequate purity of the isolated epithelia and stromal cell populations, the expression of epithelial (cadherin1, *Cdh1*) and stromal (vimentin, *Vim*) markers was evaluated by qPCR (data not shown).

### Fertility analysis

To examine the fertility of female mice, wild type (WT), *Pgr^cre/+^*, control (*Alk2^flox/flox^*), and *Alk2* cKO females were mated independently with WT fertile male mice for a 6-month period (*n* = 10 per genotype). Cages were monitored daily, and the numbers of litters and pups were recorded.

To investigate the different phases of early pregnancy we performed timed mating experiments: adult female mice were mated to fertile wild type males and the presence of vaginal plugs was monitored every morning. The morning of the plug was designated as 0.5 day post coitus (dpc); to investigate embryo attachment, 4.5 dpc pregnant animals were injected intravenously (i.v.) in the tail vein [49] with Chicago blue dye and sacrificed 10 minutes after the injection.

### Histological and immunohistochemical analysis

Uteri were dissected and fixed in 4% PFA for histology. Tissue processing and embedding in paraffin were performed in the Baylor College of Medicine Department of Pathology Core Laboratory. Paraffin sections (5 µm) were deparaffinized, rehydrated and boiled in citrate buffer for antigen retrieval. Blocking of non-specific signal was performed by incubating the sections with 10% normal goat serum (NGS) or 3% BSA in TBS-T for 1–3 hours at room temperature. Tissue sections were incubated overnight at 4°C with primary antibodies specific to ALK2 (1∶200, LifeSpan Biosciences, Inc., LS-B6835), CDH1 (1∶400, Cell Signaling, 24E10), PTGS2 (1∶1000, Thermo Scientific, RB-9072), MKI67 (1∶1000, BD BioScience, 550609), CEBPB (1∶200, Santa Cruz Biotechnology, Inc. sc-150), PGR (1∶500, DAKO, IR068) or HAND2 (1∶100, R&D Systems, AF3876). After washing with TBS-T, sections were either incubated with biotin-labeled (CDH1, PTGS2, MKI67, CEBPB, PGR, HAND2) or fluorescent-labeled (ALK2, PGR) secondary antibody for 1 hour at room temperature. Sections treated for immunohistochemistry were then incubated with VECTASTAIN ABC solution (Vector Labs) for 30 minutes followed by peroxidase substrate (Vector Labs) for signal development. Finally, sections were briefly counterstained with CAT hematoxylin (Biocare Medical), dehydrated and mounted.

Alkaline phosphatase staining was performed as previously described [50] Frozen sections were incubated with a 100 mM Tris buffer (pH 9.5) containing 5-bromo-4-chloro-3-indolyl phosphate and Nitro blue tetrazolium chloride (Roche Applied Science). Nuclear Fast Red (Vector) was used for counterstaining.

### RNA isolation, quantitative real-time PCR and microarray hybridization

Uterine tissues were collected from control and *Alk2* cKO female mice and stored immediately at −20°C until RNA extraction. Total RNA for each sample was extracted using the RNeasy Mini kit (Qiagen). Gene expression was analyzed by real-time quantitative polymerase chain reaction (qPCR). Either SYBR Green detection system (Life technologies) or TaqMan Assays-On-Demand PCR primer and probe sets (Life technologies) were used for individual gene analysis. Melt curve analysis was performed when using Sybr Green to verify a single amplification peak. Relative mRNA levels of transcript were calculated by the 2^−ΔΔC^ method after normalizing to the endogenous reference (*Rn18S*), and plotted as mean ± SEM.

For microarray analysis, we pooled RNA from three mice per genotype and performed the experiment in triplicate. Samples were tested for quality assurance on the Agilent 2100 Bioanalyzer by the Genomic and RNA Profiling Core at Baylor College of Medicine. The labeled cRNA was hybridized to Illumina Mouse WG-6 v.2.0 by the Genomics and Proteomics Core at Texas Children Hospital (Houston, TX). Array data were quantile normalized. Differentially expressed genes were defined, using two-sided t-test and fold change (on log-transformed data). Array data have been deposited on the Gene expression omnibus (GEO, accession number GSE46689).

### Human endometrial stromal cell decidualization and rhBMP2 treatment

Samples were collected from patients undergoing gynecological surgery at Ben Taub General Hospital or the Obstetrics and Gynecology Clinic of Baylor College of Medicine (Houston, TX). The study was approved by the Institutional Review Board of Baylor College of Medicine. Undifferentiated stromal cells were obtained from endometrial biopsies from normally cycling fertile women with no history of uterine disease in the proliferative phase of the cell cycle. Written informed consent was obtained before sample collection. All samples were collected under Baylor College of Medicine Institutional Review Board (IRB) approval and IRB approval from each individual hospital. After collection, samples were transported to the laboratory and processed to obtain hESC. Cells were cultured at 37°C, 5% CO_2_, 90% RH in growth medium (DMEM/F12 +10% csFBS +1% Antibiotic-Antimycotic +1% HEPES).

To investigate the role of ALK2 in controlling the decidualization of stromal cells in human we utilized siRNA specifically targeting *ALK2* to knock down the receptor (Figure S5). The day of decidualization induction was designed as day 0. During the two days prior to induction (day −2 and −1), cells in 6-well plates were treated for 6–8 hours with DMEM/F12 medium +2% csFBS (Invitrogen) containing a mix of Lipofectamine 2000 (Invitrogen) and 100 pmol of either non-targeting (NT) siRNA (ON-TARGETplus Non-targeting Control Pool, Thermo Scientific) or siRNA targeting to *ALK2* (ON-TARGETplus Mouse Acvr1 siRNA, Thermo Scientific) dissolved in Opti-MEM (Invitrogen). On day 0, an aliquot of cells for each treatment (NT or *ALK2* siRNA) was collected and the remaining cells were treated with decidual medium containing 10^−8^ M 17β-estradiol, 10^−6^ M 17α-hydroxy-6α-methylprogesterone acetate (MPA) and 50 µM cyclic AMP (cAMP) in Opti-MEM +2% csFBS +1% Antibiotic-Antimycotic (Invitrogen). The medium was changed (to freshly prepared decidual medium) on day 2, and on day 3 cells were harvested and stored at −80°C.

To investigate the effects of rhBMP2 on SMAD1,5,8 phosphorylation in human stromal cells, we dissolved rhBMP2 (R&D Systems, 355-BM) in DMEM/F12 +10% csFBS at a concentration of 100 ng/ml and we treated the cells with this solution for 30, 45, 60, 180 or 360 minutes. To test the effect of ALK2 ablation on SMAD1,5,8 phosphorylation, we performed the same experiment but transfected the cells with either NT or *ALK2* siRNA during the two days before rhBMP2 treatment as described above.

### Western blotting

After the treatment, cells were trypsinized, washed in PBS, pelleted and stored at −80°C. Protein extraction was performed by incubating the pelleted cells with RIPA buffer (Thermo Scientific) in agitation for 30–45 minutes. Protein concentration was determined by Bradford's method using BSA as the standard. Samples containing 20 µg of total protein were subjected to sodium dodecyl sulfate (SDS)-polyacrylamide gel (NuPAGE 4–12% Bis-Tris gel, Invitrogen) electrophoresis. The separated proteins were transferred onto a nitrocellulose membrane and the membrane was blocked for 1 hour with 5% skim milk in Tris-buffered saline with 10 mM Tris-HCl +150 mM NaCl +0.1% Tween 20 (TBS-T). Then, the membrane was probed overnight with antibodies against C/EBPβ (1∶500, Santa Cruz, sc-150), PGR (1∶500, Santa Cruz, sc-7208), p-SMAD1,5,8 (1∶500, Cell Signaling, 9511) or β-actin. After 1 hour-incubation with horseradish peroxidase-linked secondary antibodies, the membrane was treated with enhanced chemiluminescence reagents (SuperSignal West Pico Chemiluminescent Substrate, Thermo Scientific) to visualize the immunoreactivity.

### Chromatin immunoprecipitation (ChIP)

ChIP analysis was performed using the Imprint Chromatin Immunoprecipitation kit (Sigma) according to the manufacturer's protocol. Briefly, human endometrial stromal cells were treated with decidual medium for 3 days as described above. On the third day of treatment, cells were cross-linked in 1% formaldehyde in DMEM/F12 and after 10 minutes the reaction was stopped by adding 1.25 M glycine. Cells were washed with cold PBS, pelleted and treated with Nuclei Preparation buffer and Shearing Buffer containing protease inhibitor cocktail (PIC) to prepare the DNA for sonication. The DNA was sheared by sonication: always keeping the cells in ice, we alternated 7 cycles of 20 second sonication with one-minute periods of rest. A small aliquot of the sheared DNA was analyzed by agarose electrophoresis. Debris was precipitated by centrifugation and the supernatant was incubated with 1 µg of Normal Mouse IgG (negative control), Anti-RNA Polymerase II (positive control), anti-SMAD1/5 (Santa Cruz, sc-7965), or anti-CEBPB (Santa Cruz, sc-150) antibody overnight. The next day, crosslinking was reversed by treatment with proteinase K in Release buffer and Reversing Solution. Finally, the DNA was purified using GenElute Binding Columns. To quantify the amount of template pulled down from the ChIP reactions we performed qPCR using SYBR green detection system. We used various primer sets to amplify specific regions in the proximity of the *CEBPB* and *PGR* genes (Table S1). The putative binding sites of SMAD1/5 were found by searching the conserved consensus sequence GGCGCC (Bmp Responsive Element, BRE) in the *CEBPB* gene ±1 kb; the putative binding sites of C/EBPβ on *PGR* were identified by using http://cistrome.org/finder/.

## Supporting Information

Figure S1 Generation of *Alk2* conditional knockout mice. **A**) Illustration of the *Alk2* conditional allele with exon 7 flanked by two *loxP* sites. In cells where the promoter of progesterone receptor is activated, the *cre*-mediated recombination at the two *loxP* sites generates a conditional null allele. **B**) Male mice homozygous for the *Alk2* floxed allele (*Alk2^flox/flox^)* and carrying progesterone receptor-*cre* knock-in (*Pgr^cre/+^*) allele were bred to *Alk2^flox/flox^* female mice to generate *Alk2^flox/flox^ Pgr^cre/+^* females, which were designated as *Alk2* cKO. *Alk2^flox/flox^* female mice were used as controls. **C**) Analysis of recombination of *Alk2* floxed allele in the genomic DNA obtained from uterine tissue.
(TIF)

Figure S2 The decidualization of the uterus in *Alk2* cKO mice is significantly impaired 5 days after uterine trauma. **A–B**) Gross morphology of the uteri of *Alk2* control (**A**) and cKO (**B**) mice 5 days after the induction of decidualization. The right horn was stimulated to decidualize by scratching the luminal epithelium with a needle, while the left horn was left untreated to measure the change in weight during decidualization. **C**) Ratio of wet weight of decidual horn to wet weight of untreated horn; the uterine tissues were collected 5 days after the artificial induction of decidualization. The ratio is significantly smaller in cKO females (red column) compared to control mice (black column). (*** p<0.001). N = 5. Data are means ± SEM.
(TIF)

Figure S3 Expression of *Fkbp4* and *Fkbp5* during mouse decidualization. Expression of *Fkbp4* (**A**) and *Fkbp5* (**B**) in decidual tissue collected one day after artificial induction of decidualization was measured by qPCR. The expression of both genes is significantly lower in *Alk2* cKO mice (red column) compared to controls (black column) (* p<0.05). N = 3. Data are means ± SEM.
(TIF)

Figure S4 Progesterone receptor protein level during decidualization. Immunohistochemistry (**A–B**) and immunofluorescence (**C–D**) analysis of progesterone receptor (PGR). PGR levels are comparable in *Alk2* control (**A–C**) and cKO mice (**B–D**) during decidualization. No difference is observed in the cellular localization of progesterone receptor as well, with the protein mainly localized in the nuclei of uterine stromal cells. Epithelial cells do not show positive staining in the nuclei.
(TIF)

Figure S5 Knockdown of *Alk2* in human endometrial stromal cells. The expression of *ALK2* was measured by qPCR in cells transfected with non-targeting siRNA (black column) or with siRNA targeted to *ALK2* (red column). The percentages indicate the reduction of *ALK2* expression due to siRNA silencing. The mRNA levels of *ALK2* were quantified after siRNA transfection (day 0) and repeated after 3 days of EPC treatment. Data are means ± SEM.
(TIF)

Figure S6 *Alk2* expression during pseudopregnancy and decidualization. The expression of *Alk2* was quantified by qPCR in uteri collected at sequential time points of pseudopregnancy (**A**) and in control and decidualized horns collected one and two days after artificial induction of decidualization (**B**). Data are means ± SEM.
(TIF)

Figure S7 *Alk2* cKO mice show epithelial proliferation retention and this is not due to an alteration of Hand2. Cellular proliferation was visualized by Mki67 staining in mice treated with E2+P4 (EP) for four days (**A–B**). Expression (**C**) and protein levels (**D–E**) of HAND2 in EP-treated mice were quantified by qPCR and immunohistochemistry, respectively.
(TIF)

Figure S8 Fertility of *Alk2^f/f^ Amhr2^cre/+^* and *Alk2^f/f^ Pgr^cre/+^* mice. Comparison between the fertility of control mice and mice carrying the *cre* recombinase gene expressed under the control of *Amhr2* and *Pgr* promoters. The fertility is expressed in terms of average of total pups generated by each female (N of females for each genotype = 7–10) during the six-month fertility trial (**A**) and number of pups per litter (**B**). Data are means ± SEM.
(TIF)

Table S1 Primers for ChIP. Primer pairs used for amplifying putative binding sites of SMAD1/5 on CEBPb and of CEBPB on PGR on DNA samples processed for chromatin immunoprecipitation (ChIP).
(TIF)
