# Supplementary figures and images for: Activin-Like Kinase 2 Functions in Peri-implantation Uterine Signaling in Mice and Humans
Source: PLoS Genet. 2013 Nov 14;9(11):e1003863. doi: 10.1371/journal.pgen.1003863 (PMC3828128; doi:10.1371/journal.pgen.1003863)

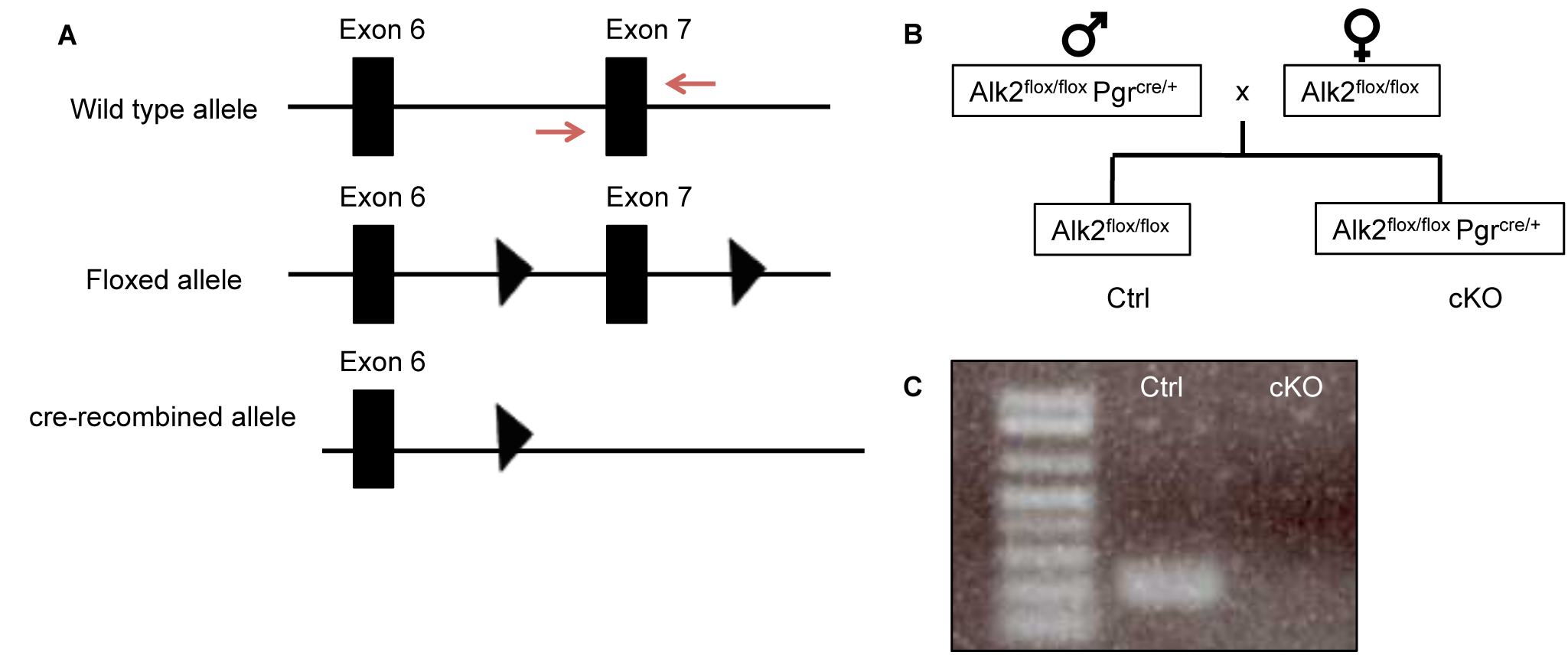

Supplement: Figure S1 — Generation of Alk2 conditional knockout mice. A) Illustration of the Alk2 conditional allele with exon 7 flanked by two loxP sites. In cells where the promoter of progesterone receptor is activated, the cre-mediated recombination at the two loxP sites generates a conditional null allele. B) Male mice homozygous for the Alk2 floxed allele (Alk2flox/flox) and carrying progesterone receptor-cre knock-in (Pgrcre/+) allele were bred to Alk2flox/flox female mice to generate Alk2flox/flox Pgrcre/+ females, which were designated as Alk2 cKO. Alk2flox/flox female mice were used as controls. C) Analysis of recombination of Alk2 floxed allele in the genomic DNA obtained from uterine tissue. (TIF) [file pgen.1003863.s001.tif]

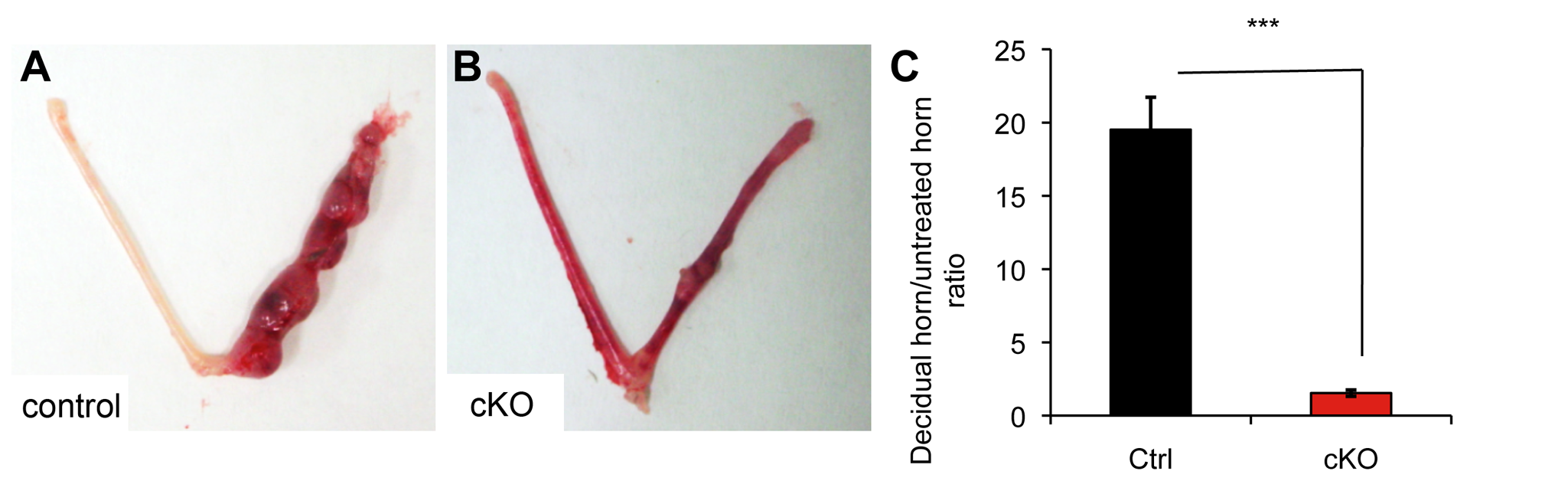

Supplement: Figure S2 — The decidualization of the uterus in Alk2 cKO mice is significantly impaired 5 days after uterine trauma. A–B) Gross morphology of the uteri of Alk2 control (A) and cKO (B) mice 5 days after the induction of decidualization. The right horn was stimulated to decidualize by scratching the luminal epithelium with a needle, while the left horn was left untreated to measure the change in weight during decidualization. C) Ratio of wet weight of decidual horn to wet weight of untreated horn; the uterine tissues were collected 5 days after the artificial induction of decidualization. The ratio is significantly smaller in cKO females (red column) compared to control mice (black column). (*** p<0.001). N = 5. Data are means ± SEM. (TIF) [file pgen.1003863.s002.tif]

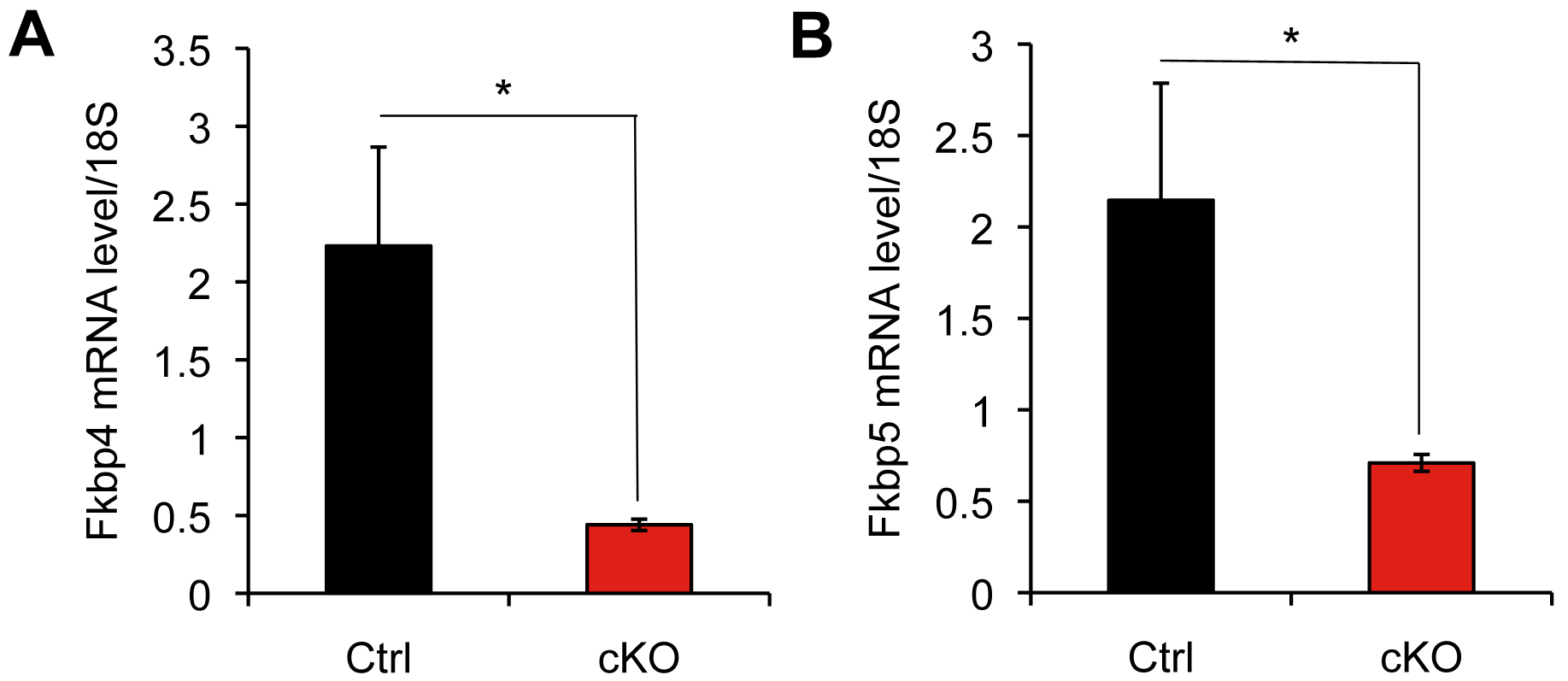

Supplement: Figure S3 — Expression of Fkbp4 and Fkbp5 during mouse decidualization. Expression of Fkbp4 (A) and Fkbp5 (B) in decidual tissue collected one day after artificial induction of decidualization was measured by qPCR. The expression of both genes is significantly lower in Alk2 cKO mice (red column) compared to controls (black column) (* p<0.05). N = 3. Data are means ± SEM. (TIF) [file pgen.1003863.s003.tif]

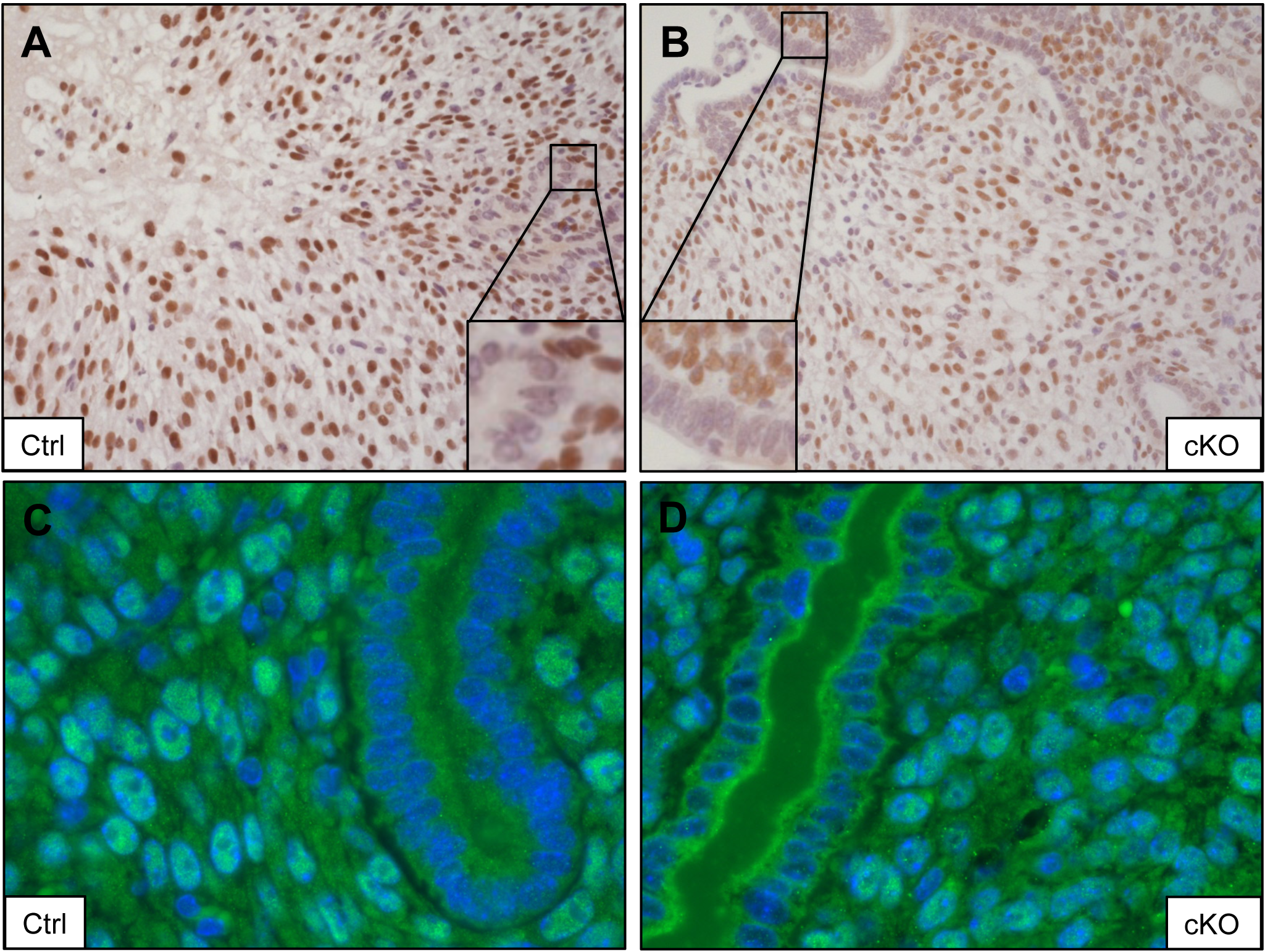

Supplement: Figure S4 — Progesterone receptor protein level during decidualization. Immunohistochemistry (A–B) and immunofluorescence (C–D) analysis of progesterone receptor (PGR). PGR levels are comparable in Alk2 control (A–C) and cKO mice (B–D) during decidualization. No difference is observed in the cellular localization of progesterone receptor as well, with the protein mainly localized in the nuclei of uterine stromal cells. Epithelial cells do not show positive staining in the nuclei. (TIF) [file pgen.1003863.s004.tif]

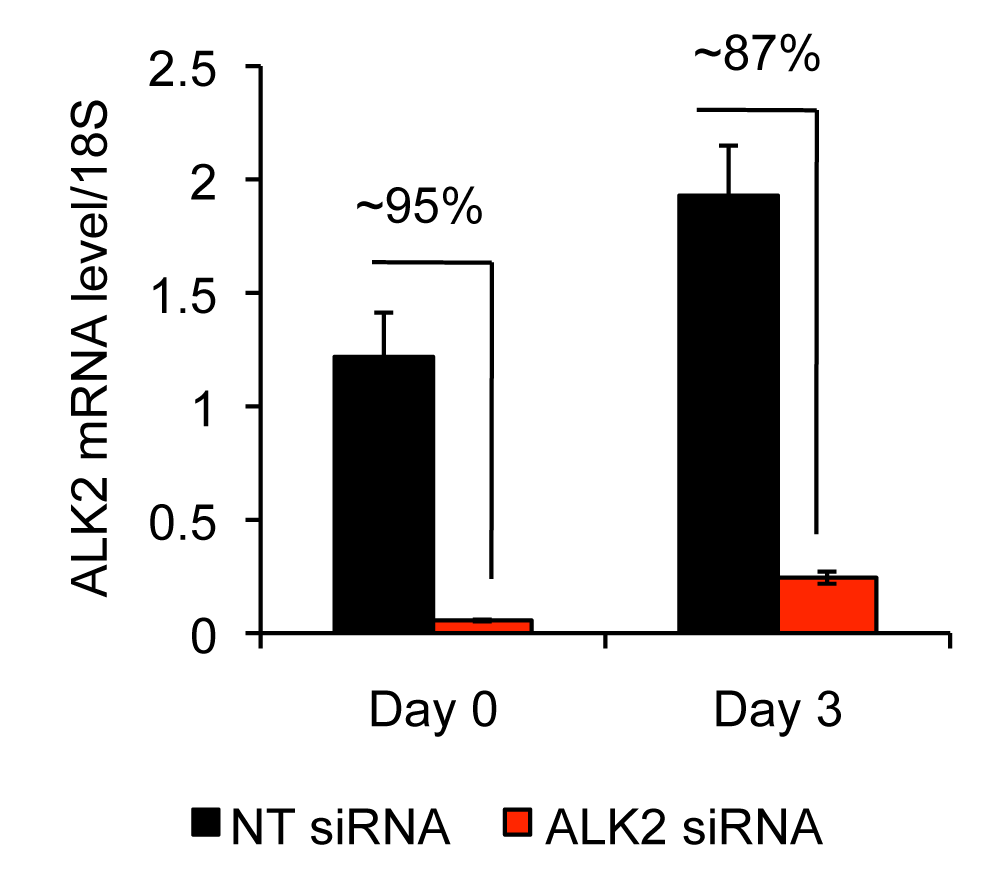

Supplement: Figure S5 — Knockdown of Alk2 in human endometrial stromal cells. The expression of ALK2 was measured by qPCR in cells transfected with non-targeting siRNA (black column) or with siRNA targeted to ALK2 (red column). The percentages indicate the reduction of ALK2 expression due to siRNA silencing. The mRNA levels of ALK2 were quantified after siRNA transfection (day 0) and repeated after 3 days of EPC treatment. Data are means ± SEM. (TIF) [file pgen.1003863.s005.tif]

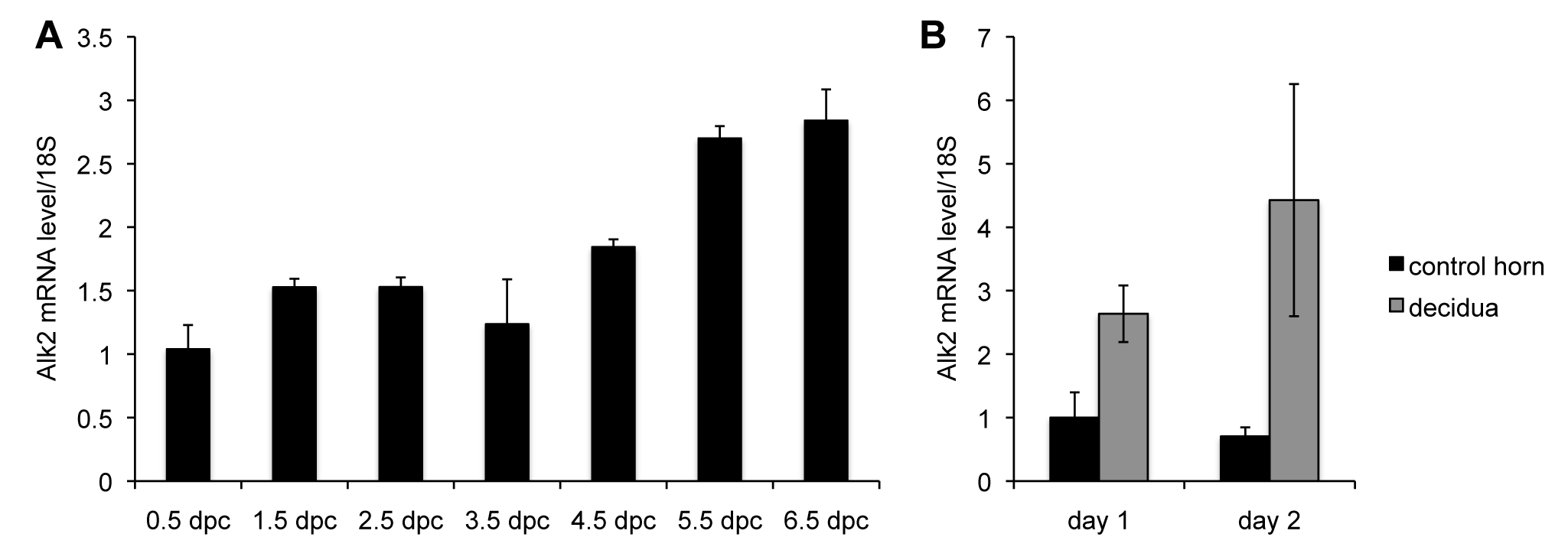

Supplement: Figure S6 — Alk2 expression during pseudopregnancy and decidualization. The expression of Alk2 was quantified by qPCR in uteri collected at sequential time points of pseudopregnancy (A) and in control and decidualized horns collected one and two days after artificial induction of decidualization (B). Data are means ± SEM. (TIF) [file pgen.1003863.s006.tif]

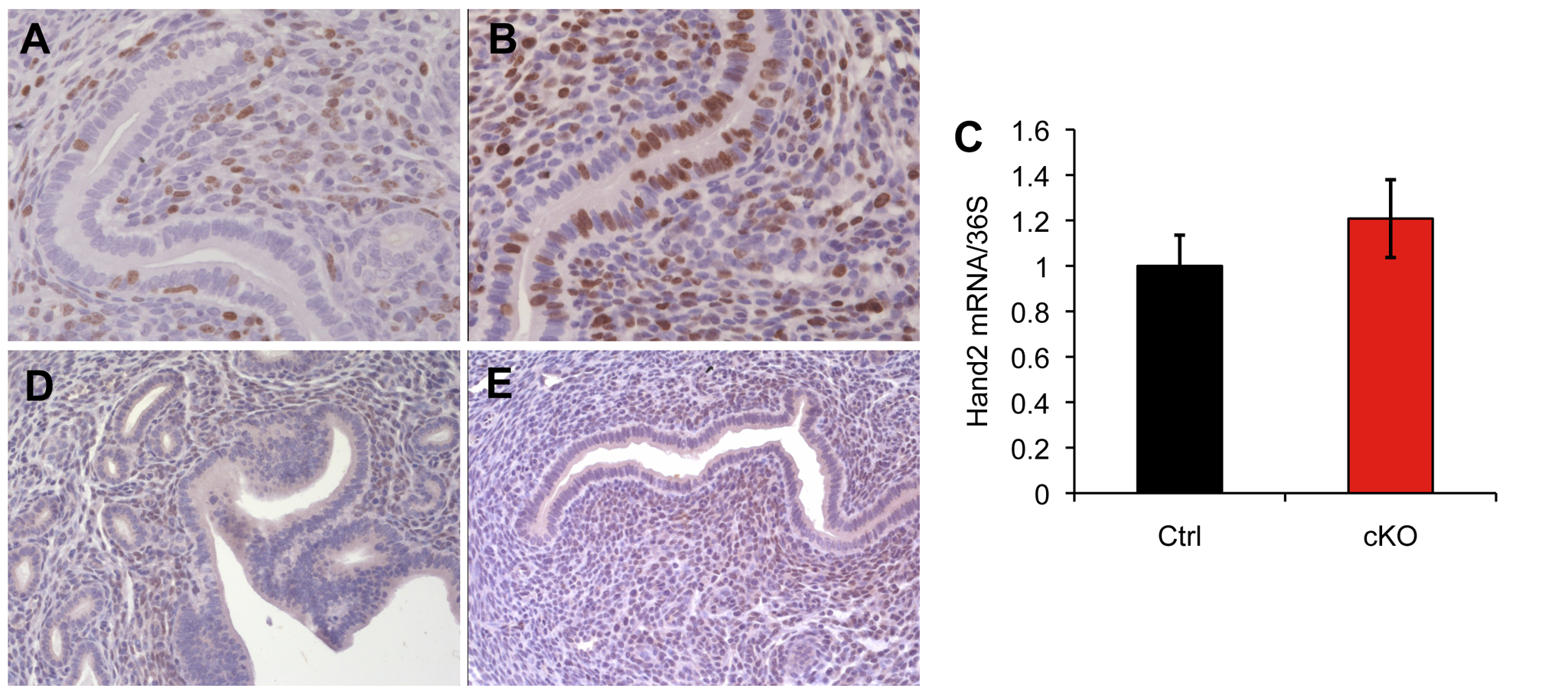

Supplement: Figure S7 — Alk2 cKO mice show epithelial proliferation retention and this is not due to an alteration of Hand2. Cellular proliferation was visualized by Mki67 staining in mice treated with E2+P4 (EP) for four days (A–B). Expression (C) and protein levels (D–E) of HAND2 in EP-treated mice were quantified by qPCR and immunohistochemistry, respectively. (TIF) [file pgen.1003863.s007.tif]

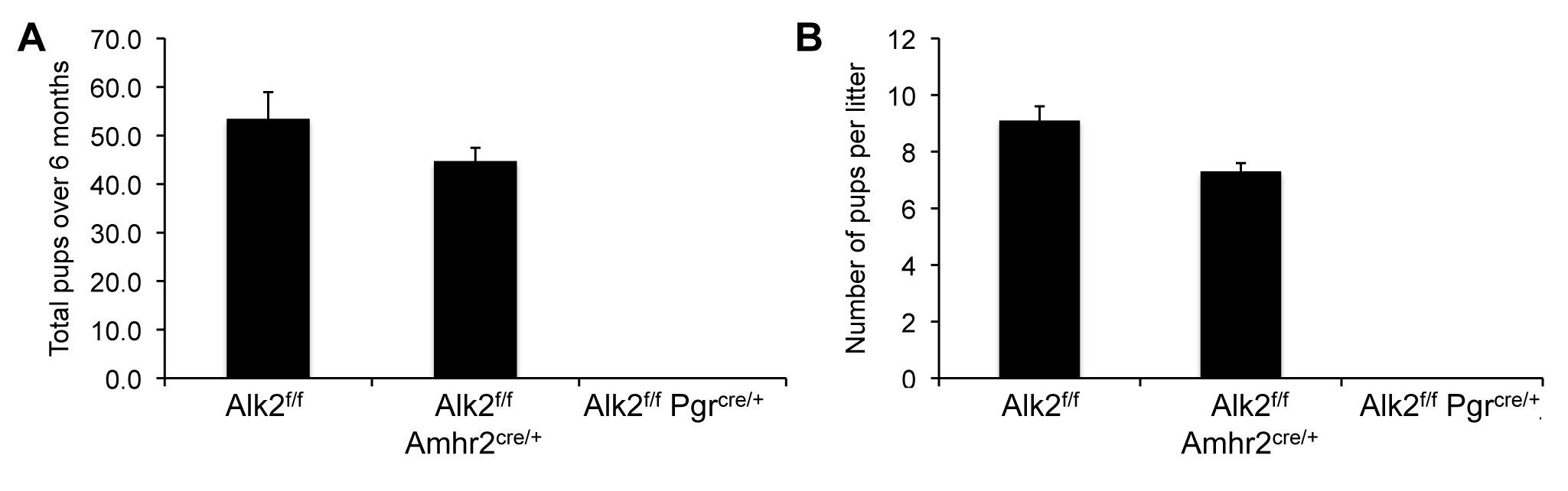

Supplement: Figure S8 — Fertility of Alk2f/f Amhr2cre/+ and Alk2f/f Pgrcre/+ mice. Comparison between the fertility of control mice and mice carrying the cre recombinase gene expressed under the control of Amhr2 and Pgr promoters. The fertility is expressed in terms of average of total pups generated by each female (N of females for each genotype = 7–10) during the six-month fertility trial (A) and number of pups per litter (B). Data are means ± SEM. (TIF) [file pgen.1003863.s008.tif]

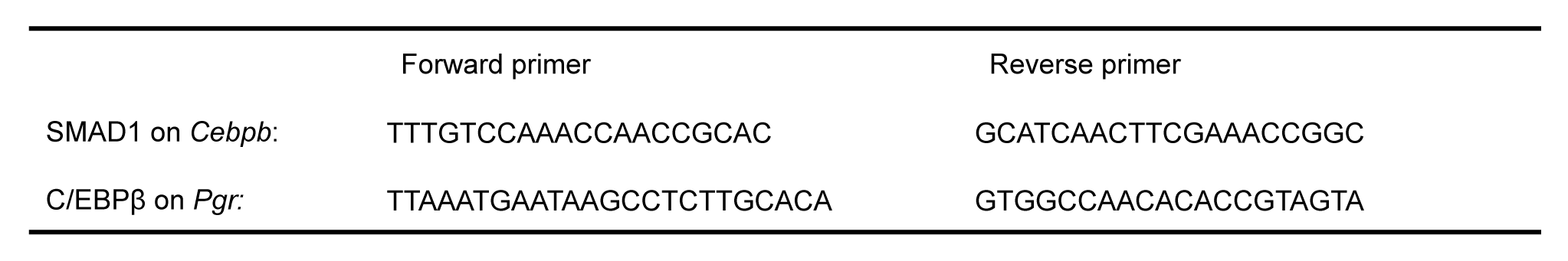

Supplement: Table S1 — Primers for ChIP. Primer pairs used for amplifying putative binding sites of SMAD1/5 on CEBPb and of CEBPB on PGR on DNA samples processed for chromatin immunoprecipitation (ChIP). (TIF) [file pgen.1003863.s009.tif]
